# COVID-19 mortality prediction in Hungarian ICU settings implementing random forest algorithm

**DOI:** 10.1038/s41598-024-62791-9

**Published:** 2024-05-24

**Authors:** Ágoston Hamar, Daryan Mohammed, Alex Váradi, Róbert Herczeg, Norbert Balázsfalvi, Béla Fülesdi, István László, Lídia Gömöri, Péter Attila Gergely, Gabor Laszlo Kovacs, Krisztián Jáksó, Katalin Gombos

**Affiliations:** 1 Department of Laboratory Medicine, Medical School, University of Pécs, Pécs, Hungary; 2 Molecular Medicine Research Group, Szentágothai Research Centre, University of Pécs, Pécs, Hungary; 3 Institute of Metagenomics, University of Debrecen, Debrecen, Hungary; 4 Department of Anaesthesiology and Intensive Care, University of Debrecen, Debrecen, Hungary; 5 Doctoral School of Neuroscience, University of Debrecen, Debrecen, Hungary; 6 Institute of Forensic Medicine, University of Debrecen, Debrecen, Hungary; 7 Department of Anaesthesiology and Intensive Care, Clinical Centre, University of Pécs, Pécs, Hungary

**Keywords:** SARS-CoV-2, COVID-19, Intensive care unit, Machine learning, Random forest, Mortality prediction, Machine learning, Viral infection

## Abstract

The emergence of newer SARS-CoV-2 variants of concern (VOCs) profoundly changed the ICU demography; this shift in the virus’s genotype and its correlation to lethality in the ICUs is still not fully investigated. We aimed to survey ICU patients’ clinical and laboratory parameters in correlation with SARS-CoV-2 variant genotypes to lethality. 503 COVID-19 ICU patients were included in our study beginning in January 2021 through November 2022 in Hungary. Furthermore, we implemented random forest (RF) as a potential predictor regarding SARS-CoV-2 lethality among 649 ICU patients in two ICU centers. Survival analysis and comparison of hypertension (HT), diabetes mellitus (DM), and vaccination effects were conducted. Logistic regression identified DM as a significant mortality risk factor (OR: 1.55, 95% CI 1.06–2.29, p = 0.025), while HT showed marginal significance. Additionally, vaccination demonstrated protection against mortality (p = 0.028). RF detected lethality with 81.42% accuracy (95% CI 73.01–88.11%, [AUC]: 91.6%), key predictors being PaO_2_/FiO_2_ ratio, lymphocyte count, and chest Computed Tomography Severity Score (CTSS). Although a smaller number of patients require ICU treatment among Omicron cases, the likelihood of survival has not proportionately increased for those who are admitted to the ICU. In conclusion, our RF model supports more effective clinical decision-making among ICU COVID-19 patients.

## Introduction

Over the past four years, SARS-CoV-2 has undergone significant evolutionary shifts in adapting to the human host, resulting in highly mutated strains with increased transmission rates. This led to the emergence of various VOCs, including Alpha, Beta, Gamma, Delta, and Omicron, which quickly became dominant regionally or globally, outcompeting previous variants. The success of each VOC over its predecessors may be due to changes in the virus’s functionality and antigenicity, which allow it to evade immune responses. The enhanced resilience of VOCs results from the interaction between virus biology and the changing landscape of human immunity, contoured by vaccination and previous infections^1^. Changes in SARS-CoV-2 strains and vaccination rates may alter various factors’ diagnostic and predictive values over time^2^, resulting in negative effects on the ICU’s clinical decision-making.

During the pandemic, highly mutated SARS-CoV-2 VOCs caused myriad complications in a great number of patients that resulted in overwhelming ICUs with cases of acute respiratory failure, sepsis, cytokine storm, multiple organ failure, thrombosis, and other complications^3–6^. This placed a massive burden and unprecedented strain on intensive care units and healthcare resources. To mitigate the huge ICU burden and reduce COVID-19 ICU mortality rates, it is crucial to not only implement a proper vaccination strategy but also ensure timely hospitalization, the right choice of therapeutic regimen, and accurate determination of disease severity^7–10^. Reportedly, the newer Omicron variant typically causes less severe illness and mortality than when compared with the Delta variant, necessitating less intensive care^11–13^. Nonetheless, a significant number of Omicron-infected patients who were administered two or three vaccine doses require intensive care, in which several patients face extremely unfavorable outcomes, ultimately expiring in or following ICU discharge.

Various wards and Intensive care units continuously generate a plethora of COVID-19 data that clinicians can not fully analyze. Hence, machine and deep learning (MDL) algorithms have been implemented for diagnosis, prognosis, therapy, public health management, and mortality prediction of COVID-19 patients prior to or following ICU admission^14–19^. However, our knowledge regarding various VOCs and their impact on ICU COVID-19 patients is incomplete, and the evolution of SARS-CoV-2 can make the application of the existing MDL models in routine clinical practice more challenging^6,20–22^. Meanwhile, recent literature lacks reflective studies with a sharp focus on well-defined COVID-19 ICU patients solely in a time span; different and newer COVID-19 lineages were confirmed circulating VOCs^2,6,20,23–27^. Therefore, utilizing a highly specific and sensitive machine learning algorithm such as RF for ICU mortality prediction using clinical and laboratory parameters along with whole genome sequencing (WGS) results of VOCs on admission day is crucial in order to improve data analysis, result interpretation, and clinical decision-making.

To the best of our knowledge, no studies explored the association of laboratory parameters in ICUs within the first 24 h of admission across different VOCs based on the results of WGS. To close the identified gap, we monitored three VOC waves in the COVID-19 ICU patients in the Southern Transdanubian region, Hungary, over a 22 month period, using WGS. We recorded the distribution of variant density among the dominant VOCs, (Alpha, Delta, and Omicron), and correlated these viral lineages with clinically documented parameters regarding the most severe cases who were admitted to the ICU to examine the relationship between viral genotypes, clinical and lab parameters, comorbidities, vaccination status, and patient lethality. Our objective was to include viral genomic data as potential biomarkers in a combined analysis to enhance prognosis and advance sensitive healthcare sectors toward improved disease progression prediction and patient risk stratification. Additionally, we have implemented RF with the requisite sensitivity and specificity to predict fatality in Hungarian ICU settings, implementing routine clinical and laboratory parameters of 649 ICU patients in two ICU centers retrospectively. The study focuses on providing interpretable insights for clinical use as this method significantly streamlines the triage process in identifying critical patients needing immediate treatment, optimizes healthcare facility workflows, and paves the way for accurate resource allocation.

## Results

### Age and gender

Notably, five hundred and three ICU patients from the Southern Transdanubian region, Hungary, were included in this study. The ICU patients were between 18 and 96 years old (median age: 66 years, IQR 57–73 years). Male patients comprised 58% of ICU admissions, while female patients accounted for the remaining 42%. The detailed age groups with gender distribution of ICU patients are depicted in Figure S1 in the Supplementary Material.

We used 2,975 SARS-CoV-2 samples of non-ICU patients from the Southern Transdanubian region, Hungary, in comparison to our 503 ICU patients. The means of age in all lineages in the ICU patients were significantly higher than when compared with those in the non-ICU patients. Male gender unveils a highly significant correlation with ICU admission in the Alpha (p < 0.001) and Delta (p = 0.005) groups, however, not the Omicron (p = 0.139) group. We categorized age into three classes: < 50 years old, 50–65 years old, and > 65 years old. In all three age groups across all lineages, a highly significant difference (p < 0.001) between ICU and non-ICU patients was observed, see Table S1 in the Supplementary Material.

### Mortality and comorbidities

Distinctively, five hundred and three patients who were afflicted with critical conditions were admitted to the ICU; subsequently, 317 out of 503 patients succumbed while in the ICU. 127 out of 194 Alpha-infected patients, amounting to 65.5%, expired in the ICU. While 152 out of 230 Delta-infected patients, amounting to 66.1%, expired in the ICU. While 38 out of the remaining 79 Omicron-infected patients, amounting to 48.1%, expired in the ICU. After following up with the ICU survivors for 28 days from the day of ICU discharge, 10 Alpha-infected patients, 17 Delta-infected patients, and 10 Omicron-infected patients succumbed following the ICU discharge in the 28 days of follow-up. See Table S2 in the Supplementary Material to explore hard mortality among all the variants.

Age was a highly significant factor in both soft and hard mortality rates in the Alpha and Delta variants, and it was significant in the case of the Omicron variant when we explored both hard and soft mortality. The most senior groups in all lineages were increasingly subjected to fatal outcome, see Figure S2 in the Supplementary Material. We performed logistic regression to check and establish a correlation between these three groups and mortality. Comorbidities such as HT, DM, and chronic obstructive pulmonary disease (COPD), had a major effect upon mortality with different magnitudes in the examined variants. HT had a highly significant effect upon mortality in the Omicron group, while DM had a significant effect upon mortality in the Delta group. Together, HT and DM had a highly significant effect upon the mortality of the Omicron-infected patients. Additionally, COPD had a significant effect upon the mortality of the Omicron-infected patients without considering inhaled corticosteroids (ICS) usage prior to ICU admission. When we explored the effect of ICS upon COVID-19 mortality, we did not notice any significant difference in the ICS group in reducing COVID-19 mortality, see Supplementary Material Table S3.

When we performed logistic regression regarding mortality, we noticed the age > 65 was highly significant in increasing the odds of mortality with an odds ratio (OR): 2.92, 95% CI: 1.68–5.12, p-value < 0.001. DM solely had a significant effect in increasing the odds of mortality (OR: 1.55, 95% CI: 1.06–2.29, p = 0.025). HT increased the odds of mortality, however, the difference was not significant (OR: 1.44, 95% CI: 0.98–2.13, p = 0.065). DM and HT did not have a significant modifier effect upon mortality odds in multivariable analysis, see Supplementary Material Table S4. Although males were dominant in all three variants in terms of mortality rates and need for intensive care, gender had no significant effect upon mortality.

### Vaccination data

In regards to vaccination, 339 out of 503 patients had no vaccination history, and among these unvaccinated patients, 222 patients (65.5%) succumbed. 35 patients received only one vaccination dose, in which 24 (68.6%) patients in this group soon after they expired. Patients with two vaccination doses following 14 days from the second vaccination dose were considered fully vaccinated; 82 patients were fully vaccinated, and 53 (64.6%) patients in this group expired. In reference to those patients who received a booster dose, 47 patients received 3 doses of vaccination, in which only 18 (38.3%) patients expired in this group. Patients were considered fully vaccinated 14 days following the second dose of vaccination, whereas fully vaccinated patients who received at least two doses of vaccination and/or at least one booster dose within a six-month window post-vaccination from the first booster dose were considered protected patients. Out of 503 patients, only 55 were deemed protected, in which 27 (49.1%) patients expired in this group.

In consideration of the 437 non-protected patients, 281 (64.3%) patients succumbed. It is noteworthy in highlighting, the remaining 11 Delta-infected patients were fully vaccinated, however, sufficient data in terms of vaccination dates were missing; 9 of these 11 patients were deceased. When we performed Pearson’s Chi-squared test, it revealed the protective effect of vaccination significantly reduces mortality among protected patients with a p-value of 0.028, see Supplementary Material Table S5.

### Follow-up of patients

We tracked the ICU survivors for a span of 28 days. Although Delta-infected ICU survivors had the lowest median age (61), out of 78 Delta-infected ICU survivors, 17 (21.8%) of these patients expired, in which the majority of the deceased were males, 6 of whom were fully vaccinated, and another 2 received boosters, however, only 3 were protected. Omicron-infected ICU survivors had the highest median age (69), 10 (24.4%) Omicron-infected ICU survivors expired even though 8 of these patients received boosters, yet only 5 of the 8 fell into our category of protected patients, in which one of these patients was fully vaccinated.

When considering the Alpha-infected ICU survivors, this group had the lowest median age (61), in which, 10 of these patients expired in the follow-up span of 28 days, only one patient was protected, and 3 others were administered partial vaccination. Some of the ICU survivors who had COPD and used ICS prior to being admitted into the ICU in all groups succumbed during this 28-day follow-up period, see Supplementary Material Table S6.

**Figure 1:**
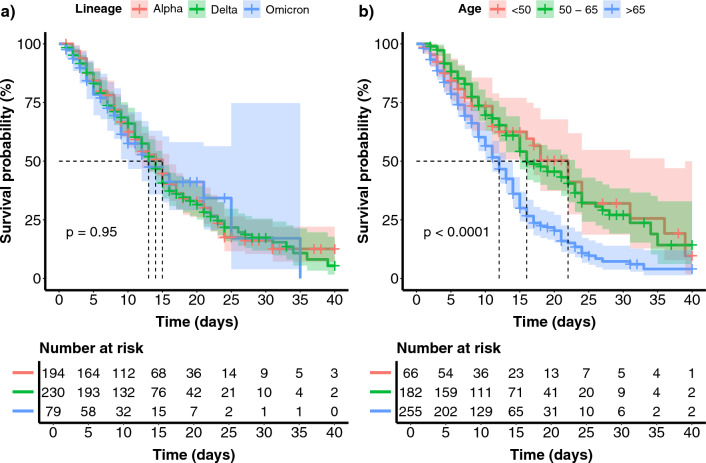
Kaplan–Meier survival probability curve. (**a**) The Kaplan–Meier survival curve shows survival probability among Alpha, Delta and Omicron VOCs. (**b**) The Kaplan–Meier survival curve shows survival probability among the age groups < 50, 50–65 and > 65 years old.

The Kaplan–Meier survival curve is one of the best options to measure the fraction of subjects living for a certain amount of time following treatment, which in our case, begins with ICU admission. Figure 1**,** panel (**a**) shows the survival curves by lineage. A 50% survival probability was reached on Day 15 in the case of Alpha-, Day 14 by Delta-, and Day 13 by Omicron patients. There was no significant difference among the lineages (p = 0.95). We also used Kaplan–Meier to compare survival probability among the three age groups (< 50, 50–65, > 65, see Fig. 1**,** panel (**b**). The youngest group of ICU patients aged < 50 years old reached a 50% survival probability on Day 22, while the middle group of patients aged between 50 and 65 years old reached a 50% survival probability on Day 16. The oldest group with age > 65 reached a 50% survival probability on Day 12. There was a very highly significant difference (p < 0.0001) among the age groups.

### Clinical and laboratory parameters among the variants

**Figure 2:**
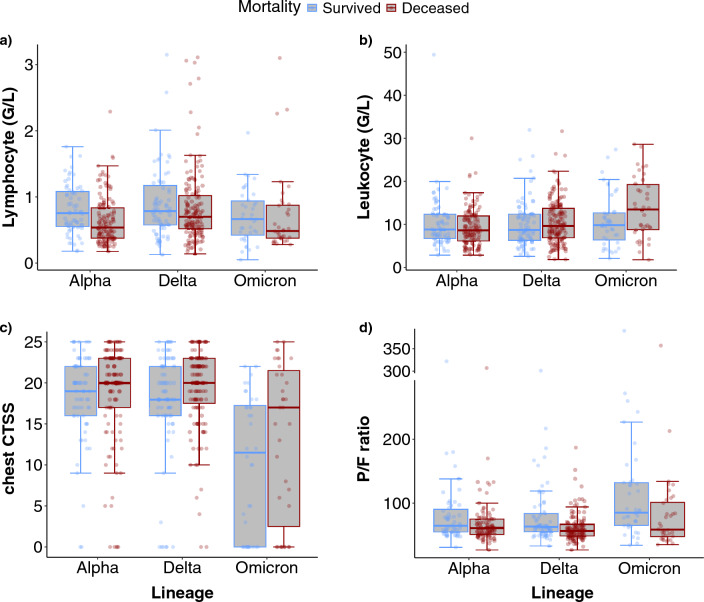
Laboratory and clinical parameters in VOCs in survivors (blue) and deceased (red) patients. (**a**) Lymphocyte count in survivors and deceased ICU patients of all lineages. (**b**) Leukocyte count in survivors and deceased ICU patients of all lineages. (**c**) Chest CTSS in survivors and deceased ICU patients of all lineages. (**d**) P/F ratio in survivors and deceased ICU patients of all lineages.

There was a significant difference between the interleukin-6 (IL-6) levels regarding the survivors and deceased patients in the group of Alpha (p = 0.006) and Delta (p = 0.008) variants, however, not in the Omicron group (p = 0.391). There was a significant difference between the ferritin levels of the survivors and deceased patients in the Delta (p = 0.009) and Omicron (p = 0.026) groups, however, not in the Alpha (p = 0.649) group. Panel (**a**), panel (**b**), panel (**c**), and panel (**d**) in Fig. 2 show (lymphocyte count, leukocyte count, chest CTSS, and P/F ratio [PaO_2_/FiO_2_ ratio, also known as Horowitz index]) in the Alpha-, Delta-, and Omicron-infected survivors survived and deceased patients, respectively. To examine IL-6, ferritin, and D-dimer in the Alpha-, Delta-, and Omicron-infected survivors and deceased patients, see Supplementary Material Figures S3- Figures S5. To explore the exact values of the laboratory and clinical parameters (IL-6, ferritin, D-dimer, lymphocyte count, chest CTSS, and P/F ratio), please see Supplementary Material Table S7.

We also amassed information in reference to the maximum level of invasiveness required for oxygenation. The number of patients who required Endotracheal Intubation (ETI) in the Alpha, Delta, and Omicron variants were 150, 183, and 50, respectively. The number of patients who required non-invasive ventilation (NIV) masks in the Alpha, Delta, and Omicron variants were 18, 24, and 13, respectively. The number of patients who required High-flow nasal cannula (HFNO_2_) in the Alpha, Delta, and Omicron variants were 16, 19, and 9, respectively. To see oxygen supplementation types in ICU patients of all lineages, see Supplementary Material **Figure S6**.

### Random forest

**Figure 3:**
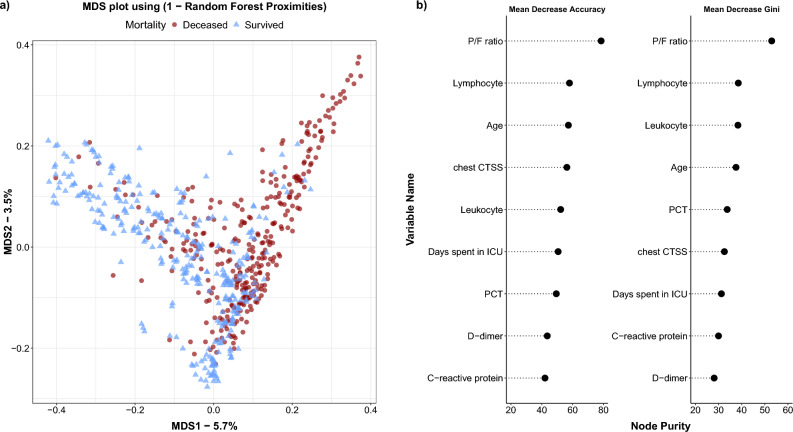
The multidimensional scaling plot along with MDA and MDG values. (**a**) MDS plot for the survivors and deceased patients. (**b**) MDA and MDG values with related levels of importance.

**Figure 4:**
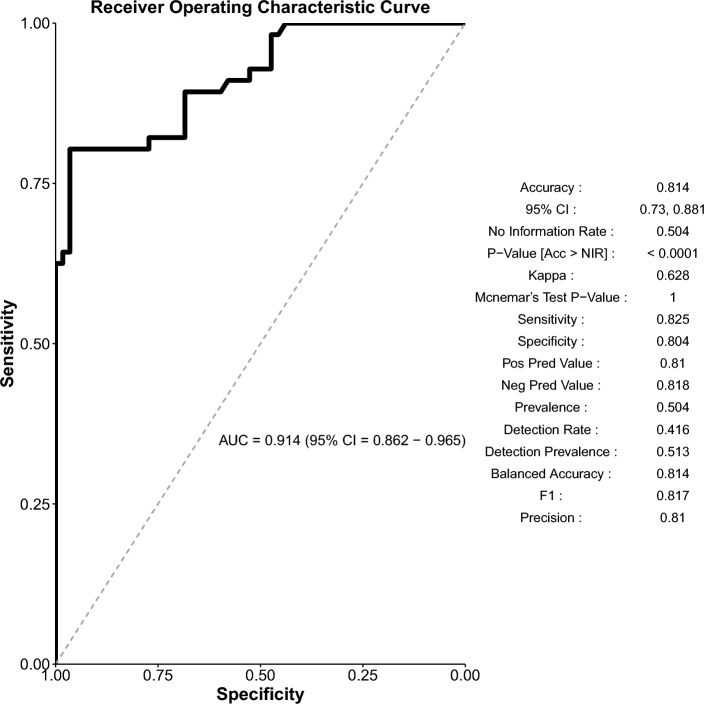
Test model performance metrics with the ROC curve.

The random forest analysis contains 649 ICU patients’ data: 503 from the original database, 22 patients infected with the lineage B.1.160, and 124 patients from the University of Debrecen. Following imputation and balancing, the model tallies 760 patients. The RF features 1000 trees, with 3 variables tried at each split. The out-of-bag estimate of error rate is 13.76%. The accuracy of the training model performance is 0.8624 (95% CI 0.8335–0.8881), with a no information rate of 0.5023 and Kappa is 0.7249, p < 2e-16. Sensitivity is 0.8523, specificity is 0.8727. More performance data are visible in Supplementary Material Figure S7. The multidimensional scaling (MDS) plot for the deceased and survivors is visible in Fig. 3, panel (**a**), while the top 9 important variables with the MeanDecreaseAccuracy (MDA) and MeanDecreaseGini (MDG) values are presented in Fig. 3, panel (**b**). The exact values of MDA and MDG can be seen in Supplementary Material Table S8. The test model performance had an accuracy of 0.814 (95% CI 0.73–0.881), with a p-value of < 0.0001. Additional performance data and the Receiver Operation Characteristics (ROC) curve are illustrated in Fig. 4.

Partial dependency plots (PDP) demonstrate the relationship between a single predictor variable and the probability of an event occurring (‘Deceased’). The most important variables of the RF (age, leukocyte count, P/F ratio, chest CTSS, and lymphocyte count) PDPs are depicted in Supplementary Material Figures S8-Figures S12.

## Discussion

SARS-CoV-2 was a viral agent that profoundly altered the global landscape; this virus, like most respiratory viruses, disproportionately affects seniors, causing severe annual outbreaks^28^. There is a growing body of evidence including epidemiological studies referencing different cohorts and meta-analyses, which demonstrates a strong association between the advanced age and comorbid burden with severe manifestation and higher mortality due to COVID-19^29,30^. While the prevalence of comorbid conditions increases with age, the relationship between age, comorbidity statements and severe or unfavorable outcomes of SARS-CoV-2 infection is complex. Ageing is a multifactorial process influenced by non-modifiable factors such as genetics, declining physiological parameters and a reduced capacity to adapt to environmental exposures. Advanced age is a significant risk factor for poor outcomes; the Centers for Disease Control and Prevention (CDC, USA) reports individuals aged 65 and above represent approximately 30% of COVID-19 infections, 45% of hospitalizations, 53% of ICU admissions and over 80% of deaths^31^. Moreover, the risk of mortality in the post-acute phase and in-hospital deaths varies by the predominant variant periods^32^.

Our retrospective analysis of the ICU confirmed the exponentially progressing correlation of mortality risk coupled together with the increase of age. Logistic regression analysis showed almost 3 times higher OR regarding the population over 65 years of age. The correlation between age and mortality was not influenced by the shifting of the predominant variant of SARS-CoV-2. However, the genetic evolution of the virus over time significantly altered the age distribution of the ICU population, with patient age changing notably during the Omicron wave. Despite males being predominant in both intensive care needs and mortality rates across all three variants, gender had no significant impact upon mortality. Additionally, the emergence of new viral lineages did not alter the correlation between gender, disease severity and adverse clinical outcome over time. Age-related comorbidities, such as HT, DM and COPD, are critical in the response mediated by SARS-CoV-2^33,34^. Our findings indicate a major effect of these comorbidities upon mortality mediated by SARS-CoV-2 variants. The presence of HT and DM in combination with age significantly affected mortality among patients infected with the Omicron variant. COPD patients on ICS prior to ICU admission were at higher mortality risk during the Omicron wave, although regular use of ICS had no significant effect upon ICU mortality.

Distinctively, we concluded while clarifying the strongest comorbidity factors, respectively, HT and DM, from age. In calculating our overall ICU population during the complete study timeline, neither DM by itself nor HT alone, or the two factors combined, yet without age, neither of these aspects lead to an increase regarding the risk of mortality. Comorbidity factors only have an effect when combined with age. Age is found to be the highest risk factor in COVID-19 infection, likely influencing all molecular mechanisms from immune responses, mitochondrial functions, endoplasmic reticulum transport mechanisms and protein folding, oxidative stress disruption, receptor activation of the ACE II and Toll-like receptors, transcription factors and cell signalling pathways^35^. Although several studies equivocally emphasize the importance of comorbidity conditions, comprehensive clinical studies are essentially needed towards evaluating their independent effect upon ICU mortality.

ICU mortality spanned a vast frontier; Carbonell et al.^36^ found it to be 28.8% during the second/third wave of the pandemic in a multicenter retrospective study (Ireland, Spain, Andorra). In Central-Eastern Europe, Benes et al.^37^ found the ICU mortality between 38.1–75.9% in the timeframe of March 2020 and February 2021. A Dutch national multicentre study^38^ reported a 28-day mortality of 35%, which increased to 43% during the follow-up at Day 90. Our findings reveal an ICU hard mortality of 63.0%, which aligns with the only Hungarian COVID-19 ICU study published to this point^39^, with an additional 7.4% increase in the 28 day follow-up period after ICU release, which leads to a soft mortality rate of 70.4%. Additionally, 10 out of 67 Alpha-infected patients, 17 out of 78 Delta-infected patients, and 10 out of 41 Omicron-infected patients expired in the 28-day follow-up, which substantiates Omicron patients contributed most to the soft mortality increase. Based on our timeline and lineage distribution, Omicron sublineages had approximately as much time (January 2022 to November 2022) when compared to Alpha and Delta combined (January 2021 to December 2021). Nevertheless, patients admitted to the ICU as a result of Omicron only represent 15.7% of total cases, which implies ICU admission was significantly lower in the case of Omicron. This can potentially be explained by the effects of protection due to vaccination and milder lineage combined. The Omicron variant is less severe; however, once admitted to the ICU, Omicron still seemingly proves to be a potentially deadly variant since there is no significant difference among the lineages in terms of survival rate, see Kaplan–Meier survival curves by lineage in Fig. 1**,** panel a).

In regards to the Hungarian population, vaccination strategies were initially introduced in December 2020. Vaccine uptake of the primary course counts up to 63.2% of the total population, with 39.8% taking up the first booster, and 4.3% in the second booster up through 4 April, 2023, in the general population^40^. Among the population admitted to the ICU in the time period of our study, 35% were unvaccinated and of those who later survived when compared to the 65% of unvaccinated patients who succumbed in COVID-19. In calculating only the full primary course, meaning the vaccinated and boosted individuals within the protective time period, vaccines are considered to have effective protection (minimum 14 days—maximum 6 months), in which we observed a significant mortality-reducing effect of the vaccination. Based on our results, we recommend those individuals over age 65 and comorbid populations will benefit from vaccination on a priority basis when compared to individuals without these conditions.

There is a widely held view Omicron is less severe in general when compared with other VOCs^12,13^. Seemingly, Omicron prefers the upper respiratory tract through the TMPRSS2-independent endosomal entry pathway, while the replication of the virus is attenuated in lower respiratory tissues^41^. This leads to a decreased rate of pulmonary invasion among Omicron patients and corresponds to a higher P/F ratio at the time of ICU admission and a lower chest CTSS when compared with Alpha and Delta VOCs, according to our study. Our study revealed ferritin levels were significantly lower among Omicron patients, which was also observed by other studies^42–44^, however, we did not find significant differences in the case of C-reactive protein (CRP), IL-6 and D-dimer, and the majority of the laboratory parameters. When we compared the survivors and deceased patients with no regard for VOCs, there were significant differences in IL-6, ferritin, lymphocyte count and D-dimer levels.

We aimed to develop a combined parameter composed of IL-6, Procalcitonin (PCT) and neutrophil/lymphocyte ratio, yet we fell short to acquire an eligible diagnostic accuracy regarding the receiver operating characteristic curve model. There are multiple and complex underlying pathobiochemical mechanisms in comorbidity and immunocompromised statements that cannot be evaluated based on a single summary laboratory result; moreover, blood samples from the patients oftentimes differ considerably during their ICU treatment.

Based on the random forest analysis, we were able to demonstrate the predictive capacity regarding the machine-learning approach in assessing ICU patient lethality due to COVID-19. The analysis—incorporating data from 649 ICU patients and employing 1000 trees with three variables used at each split to mitigate overfitting–, achieved an accuracy of 86.24% in training and 81.4% in testing phases, underscoring its robustness. Key predictors included the P/F ratio, lymphocyte count, and chest CTSS, among others, indicating the relevance of respiratory status, immune response and lung involvement in determining patient outcomes. Another key predictor of our model was the age of ICU patients, Lorenzoni et al.^45^ also reported age as the leading predictor in their models. The predictive accuracy of currently available machine learning tools ranges from 81 to 96%, based on parameters such as age, oxygen saturation, lactate dehydrogenase, urinary carbamide nitrogen, CRP, impaired kidney function, chronic kidney disease, medical history of coronary heart disease and chronic heart failure. These factors have been identified as the most influential predictors regarding COVID-19-related mortality in published literature. However, the impact of these factors upon mortality varies significantly between countries and hospitals, likely due to variations in dataset heterogeneity across study centers and distinct treatment protocols^46^.

The high accuracy and specificity of the random forest analysis^47,48^ affirm its utility in addressing a vital need in critical care medicine with early and accurate prediction of patient outcomes. The model’s predictive performance, in particular when identifying high-risk patients, can significantly impact ICU patient management strategies, emphasizing the growing role of machine learning applications in healthcare. Feature importance is vital for the identification of the most influential predictors, such as the P/F ratio, lymphocyte count, and chest CTSS, which highlights specific clinical factors that are crucial in assessing patient prognosis. These findings align with existing literature emphasizing the role of immune response, respiratory function and lung pathology in determining the severity and outcome of COVID-19 infections^49–52^.

In recent years, various MDL methods have been implemented to predict COVID-19 mortality, even in ICU settings. According to Shen^53^, the random forest model has the best performance in predicting the risk of death in hospitalized patients with COVID-19. From a technical point-of-view, the most commonly used MDL methods were random forest, logistic regression, and decision tree (commonly gradient boosted, such as XGBoost)^18^. Several studies focused on comparing the performance of multiple MDL methods^54–56^, while others focused more on the clinical point-of-view, exploring different demographic- and/or clinical- and laboratory parameters, and comorbidities in their models^57,58^. Shi et al.^59^ revealed that RF showed the best performance out of three machine learning models to predict COVID-19 mortality, with the top three important variables being mean arterial pressure, age and PCT. Sakagianni et al.^60^ also found the RF as the best outcome predictor in COVID-19 ICU patients, with urea, age, hemoglobin, CRP, platelet count and lymphocyte count as the top six important variables. Jamshidi et al.^56^ compared several machine learning algorithms to predict mortality in day 0 ICU patients, with 15 factors, mostly laboratory parameters. Random forest outperformed other models and had a superior efficiency, parameters giving the most information on the probability of each patient’s death were albumin, urea, red blood cell distribution width and age. Another Iranian study^61^ found age as the most important variable for predicting mortality in their RF analysis.

The data preprocessing, methodology, and sample sizes differ both in the listed publications and in the literature, as some include first- and second-day data or even the full length of ICU stay, some only take the laboratory parameters into consideration while others look at complex scoring systems such as SOFA or SAPS as well. Our study shows the outcome of a single RF analysis with both important clinical and laboratory parameters, and a comparison among the Alpha, Delta, and Omicron VOCs as well.

Unlike many conventional models, which may rely on a narrower set of variables, the RF approach allows for the integration of a wide range of clinical parameters, enhancing its predictive capability^46,62,63^. The practical significance of the recent study lies in its potential to support clinical decision-making by offering a model that combines high predictive accuracy with practical applicability, in which the effects of vaccination and newer VOCs are taken into consideration in a well-defined ICU population with clear exclusion criteria. In providing an accurate prediction of patient outcomes, ICU specialists can customize interventions more effectively and improve patient care and resource allocation. To cite an example, patients identified as high-risk can be prioritized for more aggressive treatment or monitoring. A further advantage regarding the RF is the model can manage non-linear relationships and interactions between variables, enhancing its predictive capability across diverse clinical scenarios. Moreover, RF is known for its resistance to overfitting, specifically with the use of multiple trees, assuring its reliability for practical applications.

Since we only had Hungarian patients’ data for model training and did not validate our result on a dataset from a different origin, it is unknown whether it is generalizable to other countries or could be only applied to the Hungarian patient population. However, we chose widely used clinical parameters (such as age, P/F ratio, and lymphocyte count). Therefore, we believe that our predictions could be useful for other researchers and clinicians as well. Another limiting factor is the model’s interpretability. While RF offers high accuracy, it is inherently more complex and less interpretable than simpler models, which may pose challenges for clinical communication, even though we tried to enhance its transparency by utilizing feature importance. The model’s performance heavily relies on the quality and completeness of input data, making it sensitive to any biases or inaccuracies present, which could be affected by the retrospective nature of the study. These strengths and limitations may underscore the potential of the random forest analysis in clinical applications while highlighting areas for improvement and careful consideration in future research and implementation.

In conclusion, when considering the application of a machine learning algorithm, our study promoted a comprehension of the mechanisms and hierarchically estimated the risk-modifying effects of demographical factors and pathophysiological and pathobiochemical parameters among patients coping with the severe course of SARS-CoV-2 infection. Advanced bioinformatical analysis regarding clinical data can potentially enable clinicians to customize guidelines and to develop care strategies and treatment alternatives, suited for the most vulnerable populations in intensive care and potentially guiding more personalized, timely interventions. Future studies can explore the integration of additional variables, such as genetic markers or detailed clinical history, in larger, externally-validated, multi-center studies with patients from diverse nations and races to refine the model’s predictive accuracy. Moreover, the application of the model in prospective clinical trials will provide valuable insights into its real-world effectiveness and impact upon patient outcomes.

## Materials and methods

### Lineage based analysis

Our retrospective study consists of a collection of 503 clinical isolates of SARS-CoV-2 with clinical parameters and laboratory biomarkers plus diagnostic values collected from 503 ICU patients in a timeframe between January 2021 through November 2022 when Alpha (B.1.1.7), Delta (B.1.617.2), and Omicron (B.1.1.529) and their sub-lineages such as (AY.4, AY.43, AY.46.6, AY.122, AY.126, AY.127, AY.129) in the case of Delta VOC, and (BA.1, BA.1.1, BA.1.17, BA.1.20, BA.2, BA.5, BA.5.2, BA.5.2.1, BE.1.1, BF.14) in the case of Omicron VOC were circulating SARS-CoV-2 lineages in the Southern Transdanubian region, Hungary. ICU patients were included who had positive SARS-CoV-2 RT-qPCR test results and were admitted due to COVID-19. Exclusion criteria were SARS-CoV-2 positive patients who were admitted to the ICU mainly due to other reasons than complications associated with COVID-19 (polytrauma, traumatic brain injury, diabetic ketoacidosis, etc.), patients with hematologic malignancies and other SARS-CoV-2 isolates such as B.1.160 (20A.EU2).

**Figure 5:**
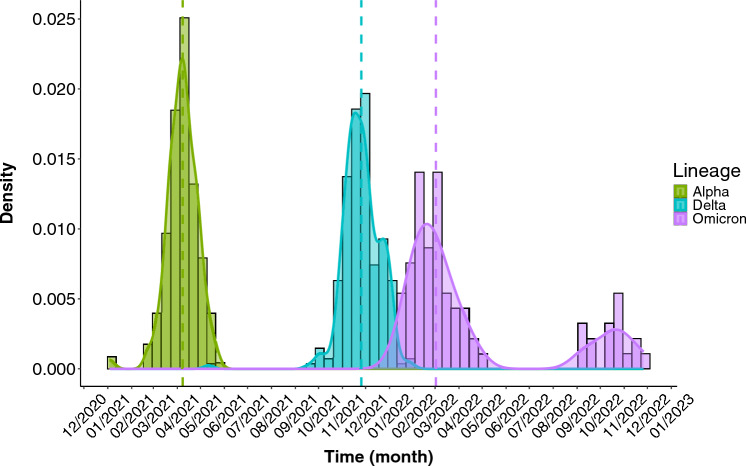
Density distribution of the different lineages. Population density distribution according to Alpha, Delta and Omicron SARS-CoV-2 VOCs recorded during the study timeline.

201 clinical isolates produced results of viral WGS, and the remaining 302 clinical isolates were included by generating a 95% range estimation based on a regional sequencing database combined with ours, totalling 2974 individuals, which was performed between 15 October 2020 up through 14 February 2022. Originally, we began with 510 ICU patients, however, seven patients were excluded in the transition phase of Delta to Omicron, since the range estimation was not possible due to the overlap between the two variants in early January 2022, see Fig. 5. The patients were confirmed by having a SARS-CoV-2 RT-qPCR positive test, in which several were tested more than once due to follow-up purposes.

This study aimed to correlate the result of partial WGS and 95% range estimation of the virus to the assessed clinical outcomes using several surrogate endpoints and biomarkers of both internal medicine such as [CRP, PCT, ferritin, IL-6, lymphocyte count, neutrophil count, granulocyte count, leukocyte count, and D-dimer] and ICU databases, chest CTSS, clinical documentation of the cases, and records of the ICU database including primary O_2_ saturation, type of O_2_ supplementation, Horowitz index (P/F ratio, first and lowest within the first 24 h of ICU admission), length of stay in ICU, final clinical endpoint, and follow-up mortality or soft mortality. Hard mortality can be translated as pure ICU mortality, and soft mortality can be interpreted as ICU mortality plus mortality outside of the ICU within 28 days of follow-up.

### Random forest analysis

**Figure 6:**
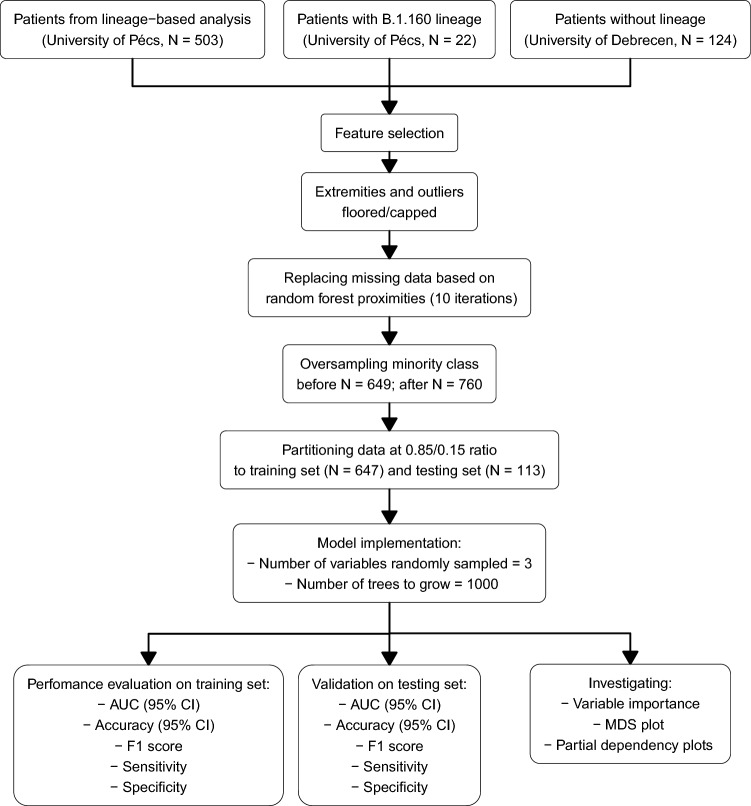
Workflow chart of the random forest analysis.

The final chapter of our results section includes a machine learning algorithm-based analysis referred to as RF, which covers an additional 22 patients from the ICU of the University of Pécs Clinical Centre with early European lineage (B.1.160) in addition to our previous database with 503 individuals, including 124 more patients with unidentified lineages from ICUs of Debrecen University, adding up to 649 total patients. The database from the University of Debrecen was assembled according to the same structure as described in the lineage-based analysis. RF classifier was applied to predict the ICU mortality of SARS-COV-2 infected patients. The workflow is depicted below in Fig. 6. In our RF model, we used nine parameters: Age, P/F ratio, chest CTSS, Days at ICU, D-dimer, PCT, CRP, Leukocyte count, Lymphocyte count as predictive features and ICU mortality as a binary outcome (Deceased, Survivor). We selected predictors based on their potential importance and to account for multicollinearity, excluding those which are highly correlated with one another. Since several parameters experienced large numbers of missing data points, for example, up to 16% in the case of the Horowitz index, we used data imputation prior to model fitting. To do so, we used the rfImpute function from randomForest (v4.7–1.1)^64,65^ R package in 649 ICU patients’ data. However, all data imputation methods should be used with scrutiny, since it can introduce potential bias into the analyses. Therefore, we applied a sensitivity test following each data curation step. We compared the original data with the after imputation and after over-sampling datasets for each of the used laboratory parameters, to investigate if there was any significant change in distribution and mean or median values. We applied the Kruskal–Wallis rank sum test for continuous or Pearson’s Chi-squared test in the case of categorical variables. We did not observe any significant bias due to data imputation (Supplementary Material Table S9).

The rfImpute function uses a proximity matrix from the RF model to impute missing values in predictive variables. The algorithm initiates by imputing either the medians or modes, then proceeds to fit the RF model to the now complete data. Subsequently, it utilizes the proximity matrix to enhance the imputation of missing values. This iterative process continues for a preset number of iterations. For continuous variables, the weighted average of the non-missing observations and for categorical predictors, the largest average proximities are imputed. We iterated 10 times the data imputations, each time using 500 trees. Since the mortality outcome was moderately imbalanced (deceased = 0.59/survivor = 0.41), we oversampled the minority class (survivors) to achieve a 0.5 ratio between classes with the use of “ovun.sample” function from the ROSE (v0.0.4) R package^66^. The function generates new samples of synthetic data by enlarging the feature space of minority and majority class examples, which are drawn from a conditional kernel density estimate of the two classes. The methods are described in detail by Menardi and Torelli^67^. After oversampling, we acquired 760 data points. Furthermore, with the increase of sample size, we could improve our estimation of mortality. Then the data were partitioned randomly to a training and testing set in 0.85/0.15 ratio (training data, N = 647, testing data, N = 113). Through the use of this dataset, we made a bagging classifier random forest (RF) model using randomForest from randomForest (v4.7–1.1) R package, which is based on Breiman’s algorithm^64^. During tree building, three features are randomly selected for data splitting at each split, for model construction, 1000 trees have been built. In the case of each new tree, a bootstrap approach has been used, and a new training set is drawn with a replacement from the original balanced and NA replaced training set. Through the use of this method, approximately 36% of the built trees do not use all training data points^68^.

After model fitting, we investigated the RF model performance on the test dataset, defining the most widely used metrics: accuracy with 95% confidence intervals (CI), sensitivity, specificity, and area under the curve (AUC). To calculate these values we used the model predictions to predict new data of the training and testing datasets. Next, in a 2 × 2 confusion matrix, we compared the predictions with the actual data. Through the use of this approach we investigated how well the model performed on supplied training data and also how it performed regarding previously unseen data. To monitor generalization error of the model, the out-of-bag error has been used. Furthermore, we calculated 95% confidence intervals with an exact method for the AUC and accuracy metrics, therefore, we have far more than merely a point estimate. Using the 1-proximity matrix of the RF model, we have completed classic multidimensional scaling (MDS)^69,70^. Based on the mean decrease in the Gini score and upon model accuracy, we determined the order of variable importance as well.

### Statistics and data management

Patient clinical and demographic data were originally documented in the local hospital information systems (e-MedSolution {T-Systems, Hungary}, IntelliSpace Critical Care and Anesthesia [ICCA] {Philips Medical Systems, USA}). The viral genomic results were originally stored in the Genomics and Bioinformatics Core Facility, Szentágothai Research Centre. Our extracted data was registered using Excel 2015 (Microsoft, Redmond WA, USA). The final manual database includes an anonymized ID from name and insurance number. The database also contained information regarding the following: gender, age, date and place of testing, RT-qPCR test results with the cycle threshold values, viral WGS results, days spent in ICU, chest CTSS which is calculated based on the percentage of lung lobe involvement [1–5 severity score added up per lung lobe, maximum score is 25], mortality, vaccination history, P/F ratio with two columns, one with the first value following ICU admission, and one with the lowest value during the first 24 h of ICU stay, follow-up of the ICU survivors for 28 days following ICU discharge, laboratory parameters in the first 24 h of ICU stay such as CRP, PCT, ferritin, IL-6, lymphocyte-, leukocyte-, neutrophil-, and granulocyte count, D-dimer, history of the comorbidities like HT, DM, COPD [with differentiating those who used inhalational corticosteroids prior to ICU admission], chronic kidney disease (CKD), immunosuppression, malignancy). An upper limit was applied for CRP, PCT, ferritin, IL-6 and D-dimer based on the first limit of laboratory measurements. These interventions did not cause any differences in any of the statistical significances, however, created an opportunity to design clearer figures and tables with less distortion by outlier values. All statistical calculations were performed in R Statistics version 4.3.2 (R Foundation for Statistical Computing, Vienna, Austria).

In regards to descriptive statistics, we reported frequencies and percentages for categorical variables, or mean with standard deviation (SD), median with interquartile range (IQR) and minimum and maximum values for continuous variables. In tables of descriptive statistics for each variable, we offer the above-mentioned statistics separately for every comparison group and also an overall summary statistic which represents our sampled population. Additionally, the corresponding p-value for the applied statistical test is given. The chi-square test or Fisher’s exact test has been used to investigate independence between two categorical variables. In the case of continuous variables, we used the Wilcoxon rank sum test to assess the difference between the medians of the two groups. One-way ANOVA and Pearson’s Chi-squared tests were applied to compare the parameters of ICU and non-ICU patients in the case of each lineage separately. We also used univariate and multivariate logistic regression models to investigate the effects of different parameters on mortality. We provided the resulting odds ratios (OR) and their 95% confidence intervals, calculated with the Wilson/Brown method^71^. Furthermore, Kaplan–Meier curves have been used to investigate how the different age groups or lineages are affecting patients’ survival probability. To statistically compare differences in survival curves, log-rank tests have been used. A p-value < 0.05 was defined as a two-tailed level of significance.

### Ethical approval

No informed consent was required due to the retrospective nature of the study and anonymized data curation. Data was collected retrospectively after ethical approval had been given by both the University of Pécs Clinical Centre Regional Committee for Research Ethics (under reference numbers KK/978-1/2022 and 9429-PTE 2022), and the University of Debrecen Clinical Centre Regional Committee for Research Ethics (under reference number DE RKEB/IKEB 6594-2023).

### Informed consent statement

Patients’ data were used retrospectively and anonymously, so after the Hungarian legal regulation informed consent was not required, according to the decision of the Local and Regional Research Ethics Committee under reference number 9429–PTE 2024.

### Institutional review board statement

Data was collected retrospectively after ethical approval had been given by both the University of Pécs Clinical Centre Regional Committee for Research Ethics (under reference numbers KK/978-1/2022 and 9429-PTE 2022), and the University of Debrecen Clinical Centre Regional Committee for Research Ethics (under reference number DE RKEB/IKEB 6594-2023).

### Supplementary Information

Supplementary Information.

## Data Availability

Source data are provided with this article. Due to the sensitive nature of the data, all the clinical datasets generated during and/or analyzed during the current study are available from the corresponding author upon request (K.G.).
