# Supplementary material for: COVID-19 mortality prediction in Hungarian ICU settings implementing random forest algorithm
Source: Sci Rep. 2024 May 24;14:11941. doi: 10.1038/s41598-024-62791-9 (PMC11126653; doi:10.1038/s41598-024-62791-9)
Supplement: Supplementary file 1 — Supplementary Information. [file 41598_2024_62791_MOESM1_ESM.pdf]

COVID-19 mortality prediction in Hungarian ICU settings implementing random forest algorithm

Ágoston Hamar[1,2], Daryan Mohammed[2], Alex Váradi[2,3], Róbert Herczeg[2], Norbert Balázsfalvi[4], Béla Fülesdi[4], István László[4], Lídia Gömöri[5], Péter Attila Gergely[6], Gabor Laszlo Kovacs[1,2], Krisztián Jáksó[7†] and Katalin Gombos[1,2†*]

[1] Department of Laboratory Medicine, Medical School, University of Pécs, Pécs, Hungary
[2] Molecular Medicine Research Group, Szentágothai Research Centre, University of Pécs, Pécs, Hungary
[3] Institute of Metagenomics, University of Debrecen, Debrecen, Hungary
[4] Department of Anaesthesiology and Intensive Care, University of Debrecen, Debrecen, Hungary
[5] Doctoral School of Neuroscience, University of Debrecen, Debrecen, Hungary
[6] Institute of Forensic Medicine, University of Debrecen, Debrecen, Hungary
[7] Department of Anaesthesiology and Intensive Care, Clinical Centre, University of Pécs, Pécs, Hungary

Emails: hamaragoston@gmail.com, daryan.mohammed@pte.hu, varadi.alex@pte.hu, herczeg.robert@pte.hu, balazsfalvinorbert@gmail.com, fulesdi@med.unideb.hu, lacipityu@gmail.com, gomori.lidia@med.unideb.hu, gergely.peter@med.unideb.hu, kovacs.l.gabor@pte.hu, jakso.krisztian@pte.hu

[†] Krisztián Jáksó and Katalin Gombos jointly supervised the work

* Correspondence: Gombos.katalin@pte.hu

Supplementary materials:

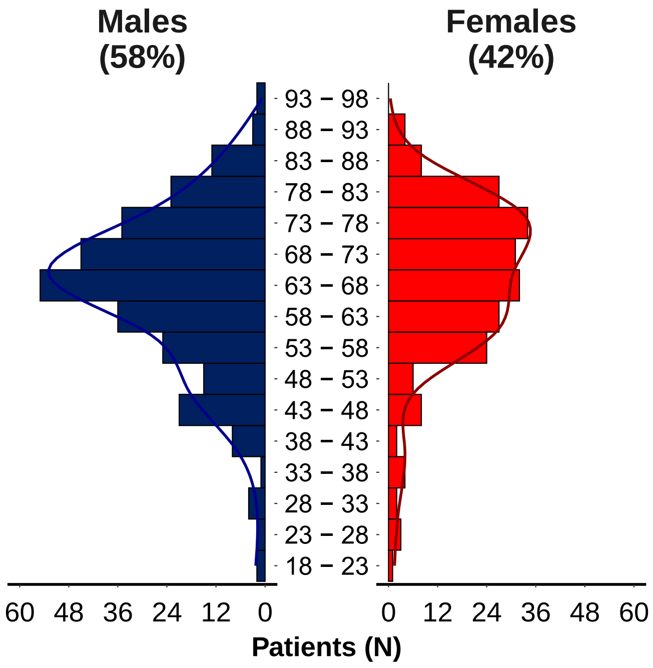

Figure S1. Age and gender of ICU patients

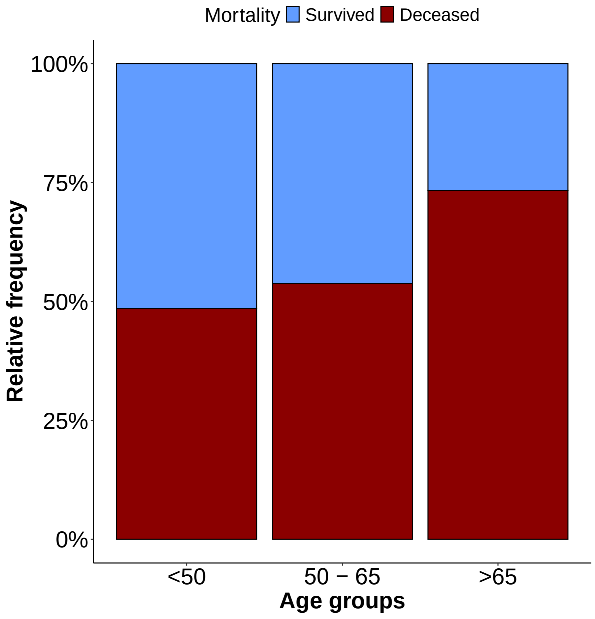

Figure S2. Relative frequency of mortality in different age groups of ICU patients

Table S1. Age and gender distribution among SARS-CoV-2 lineages of the COVID-19 patients

	**Alpha**, N = 2001				**Delta**, N = 685				**Omicron**, N = 792			
**Variables**	**Overall, N = 2,001**	**ICU, N = 194**	**Non-ICU, N = 1,807**	**p-value**	**Overall, N = 685**	**ICU, N = 230**	**Non-ICU, N = 455**	**p-value**	**Overall, N = 792**	**ICU, N = 79**	**Non-ICU, N = 713**	**p-value**
**Age**				**<0.001[1]**				**<0.001[1]**				**<0.001[1]**
Mean (SD)	52 (20)	64 (13)	51 (21)		52 (21)	63 (13)	46 (21)		42 (22)	70 (14)	39 (20)	
Median (IQR)	53 (38, 67)	65 (56, 73)	51 (36, 66)		56 (36, 68)	65 (58, 72)	44 (30, 62)		43 (25, 59)	71 (62, 81)	40 (22, 53)	
Minimum; Maximum	0; 101	25; 90	0; 101		0; 93	17; 90	0; 93		0; 96	20; 96	0; 92	
N (% not missing)	2,001 (100)	194 (100)	1,807 (100)		685 (100)	230 (100)	455 (100)		792 (100)	79 (100)	713 (100)	
**Gender**				**<0.001[2]**				**0.005[2]**				0.139[2]
Male	964 (48.2 %)	117 (60.3 %)	847 (46.9 %)		341 (49.8 %)	132 (57.4 %)	209 (45.9 %)		349 (44.1 %)	41 (51.9 %)	308 (43.2 %)	
Female	1,037 (51.8 %)	77 (39.7 %)	960 (53.1 %)		344 (50.2 %)	98 (42.6 %)	246 (54.1 %)		443 (55.9 %)	38 (48.1 %)	405 (56.8 %)	
**Age group, N**				**<0.001[2]**				**<0.001[2]**				**<0.001[2]**
<50	927 (46.3 %)	27 (13.9 %)	900 (49.8 %)		305 (44.5 %)	32 (13.9 %)	273 (60.0 %)		518 (65.4 %)	7 (8.9%)	511 (71.7 %)	
50 - 65	502 (25.1 %)	71 (36.6 %)	431 (23.9 %)		185 (27.0 %)	91 (39.6 %)	94 (20.7 %)		139 (17.6 %)	20 (25.3 %)	119 (16.7 %)	
>65	572 (28.6 %)	96 (49.5 %)	476 (26.3 %)		195 (28.5 %)	107 (46.5 %)	88 (19.3 %)		135 (17.0 %)	52 (65.8 %)	83 (11.6 %)	

[1] One-way ANOVA; [2]Pearson's Chi-squared test.

Table S2. Age and gender distribution among lineages in ICU patients

**Variables**	**Alpha**, N = 194				**Delta**, N = 230				**Omicron**, N = 79			
	**Overall**, N = 194	**Survived**, N = 67	**Deceased**, N = 127	**p-value**	Overall, N = 230	Survived, N = 78	Deceased, N = 152	**p-value**	**Overall**, N = 79	**Survived**, N = 41	**Deceased**, N = 38	**p-value**
**Age**				**0.001[1]**				**<0.001[1]**				0.073[1]
Mean (SD)	64 (13)	60 (13)	66 (13)		63 (13)	59 (13)	65 (12)		70 (14)	67 (15)	73 (12)	

Median (IQR)	65 (56, 73)	61 (54, 67)	69 (57, 74)		65 (58, 72)	61 (54, 69)	67 (60, 74)		71 (62, 81)	69 (60, 77)	76 (66, 82)	
Minimum; Maximum	25; 90	25; 90	29; 88		18; 90	18; 84	18; 90		20; 96	20; 95	40; 96	
**Age group**				**<0.001[2,3]**				**<0.001[2,3]**				0.089[2,3]
<50	27 (13.9%)	11 (16.4%)	16 (12.6%)		32 (13.7%)	17 (21.5%)	15 (9.7%)		7 (8.9%)	6 (14.6%)	1 (2.6%)	
50 - 65	71 (36.6%)	39 (52.2%)	36 (28.3%)		91 (39.6%)	37 (47.4%)	54 (35.5%)		20 (25.3%)	12 (29.3%)	8 (21.1%)	
>65	96 (49.5%)	21 (31.3%)	75 (59.1%)		107 (46.5%)	24 (30.8%)	84 (54.6%)		52 (65.8%)	23 (56.1%)	29 (76.3%)	
**Gender**				0.855[3]				0.947[3]				0.745[3]
Male	117 (60.3%)	41 (61.2%)	76 (59.8%)		132 (57.4%)	45 (57.7%)	87 (57.2%)		41 (51.9%)	22 (53.7%)	19 (50.0%)	
Female	77 (39.7%)	26 (38.8%)	51 (40.2%)		98 (42.6%)	33 (42.3%)	65 (42.8%)		38 (48.1%)	19 (46.3%)	19 (50.0%)	

[1] Wilcoxon rank sum test
[2] Fisher's Exact Test for Count Data with simulated p-value (based on 2000 replicates)
[3] Pearson's Chi-squared test

Table S3. Comorbidities among lineages in ICU patients

**Comorbidities**	**Alpha**, N = 194				**Delta**, N = 230				**Omicron**, N = 79			
	**Overall,** N = 194	**Survived,** N = 67	**Deceased,** N = 127	**p-value**	**Overall**, N = 230	**Survived,** N = 78	**Deceased,** N = 152	**p-value**	**Overall,** N = 79	**Survived,** N = 41	**Deceased,** N = 38	**p-value**
**Hypertension, N (%)**	134 (69.1%)	47 (70.1%)	87 (68.5%)	0.814[3]	162 (70.4%)	51 (65.4%)	111 (73.0%)	0.229[3]	56 (70.9%)	23 (56.1%)	33 (86.8%)	**<0.003[3]**
**Diabetes mellitus, N (%)**	74 (38.1%)	24 (35.8%)	50 (39.4%)	0.628[3]	89 (38.7%)	23 (29.5%)	66 (43.4%)	**0.040[3]**	23 (29.1%)	10 (24.4%)	13 (34.2%)	0.337[3]
**HT and DM**				0.922[2]				0.193[2]				**0.023[2]**
None	51 (26.3%)	18 (26.9%)	33 (26.0%)		59 (25.7%)	25 (32.1%)	34 (22.4%)		21 (26.6%)	16 (39.0%)	5 (13.2%)	

Both	65 (33.5%)	22 (32.8%)	43 (33.9%)		80 (34.8%)	21 (26.9%)	59 (38.8%)		21 (26.6%)	8 (19.5%)	13 (34.2%)	
Diabetes	9 (4.6%)	2 (3.0%)	7 (5.5%)		9 (3.9%)	2 (2.6%)	7 (4.6%)		2 (2.5%)	2 (4.9%)	0 (0.0%)	
Hypertension	69 (35.6%)	25 (37.3%)	44 (34.6%)		82 (35.7%)	30 (38.5%)	52 (34.2%)		35 (44.3%)	15 (36.6%)	20 (52.6%)	
**COPD, N (%)**	16 (8.2%)	5 (7.5%)	11 (8.7%)	0.773[3]	31 (13.5%)	9 (11.5%)	22 (14.5%)	0.537[3]	18 (22.8%)	13 (31.7%)	5 (13.2%)	**0.057**[3]
**COPD-ICS, N (%)**	6 (3.1%)	3 (4.5%)	3 (2.4%)	0.418[3,4]	18 (7.8%)	6 (7.7%)	12 (7.9%)	0.957[3,4]	12 (15.2%)	9 (22.0%)	3 (7.9%)	0.082[3,4]

[2] Fisher's Exact Test for Count Data with simulated p-value (based on 2000 replicates).
[3] Pearson's Chi-squared test.
[4] Fisher's exact test.

Table S4. Logistic regression analysis on age and comorbidities in ICU patients

**Characteristic**	**Univariable**					**Multivariable**				
	**N**	**Event N**	**OR[1]**	**95% CI[2]**	**p-value**	**N**	**Event N**	**OR[1]**	**95% CI[2]**	**p-value**
Age group	503	317				503	317			
<50	66	32	—	—		66	32	—	—	
50 - 65	182	98	1.24	0.71, 2.18	0.5	182	98	1.17	0.65, 2.11	0.7
>65	255	187	2.92	1.68, 5.12	**<0.001**	255	187	2.70	1.48, 4.95	**<0.001**
Diabetes mellitus	503	317								
No	317	188	—	—						
Yes	186	129	1.55	1.06, 2.29	**0.025**					
Hypertension	503	317								
No	151	86	—	—						
Yes	352	231	1.44	0.98, 2.13	0.065					
Gender	503	317				503	317			
Male	290	182	—	—		290	182	—	—	
Female	213	135	1.03	0.71, 1.48	0.9	213	135	0.94	0.64, 1.38	0.8
HT and DM	503	317				503	317			
None	131	72	—	—		131	72	—	—	
Both	166	115	1.85	1.15, 2.99	**0.012**	166	115	1.39	0.83, 2.34	0.2
Diabetes	20	14	1.91	0.72, 5.68	0.2	20	14	2.01	0.74, 6.06	0.3
Hypertension	186	116	1.36	0.86, 2.14	0.2	186	116	1.12	0.69, 1.82	0.6

[1] OR = Odds Ratio
[2] CI = Confidence Interval

Table S5. Mortality data among non-vaccinated and vaccinated ICU patients

Variables	Vaccinated				Total	p-value	Protected		Total	p-value
	**Not**	**Partially**	**Fully**	**Booster**			No	Yes		
Mortality						**0.003**[1]				**0.028**[1]
**Survived**	117 (35%)	11 (31%)	29 (35%)	29 (62%)	186 (37%)		156 (36%)	28 (51%)	184 (37%)	
**Deceased**	222 (65%)	24 (69%)	53 (65%)	18 (38%)	317 (63%)		281 (64%)	27 (49%)	308 (63%)	
Total, n (%)	339 (100%)	35 (100%)	82 (100%)	47 (100%)	503 (100%)		437 (100%)	55 (100%)	492 (100%)	
[1] Pearson's Chi-squared test										

Table S6. Detailed characteristics of ICU survivor and post-ICU mortality

**Variables**	**Alpha ICU Survivors** N = 67	**Delta ICU Survivors** N = 78	**Omicron ICU Survivors N = 41**
**Age of ICU Survivors** (median)	61	61	69
**Age of deceased patients in the follow-up** (median)	69	65	69
**Post-ICU mortality** (28 days follow-up, number of patients)	10	17	10
**Protected patients**	2	9	17
**Gender** (Male/Female ratio)	41/26	45/33	22/19
**Mortality in COPD-ICS users** (deceased/total)	1/3	1/6	6/9

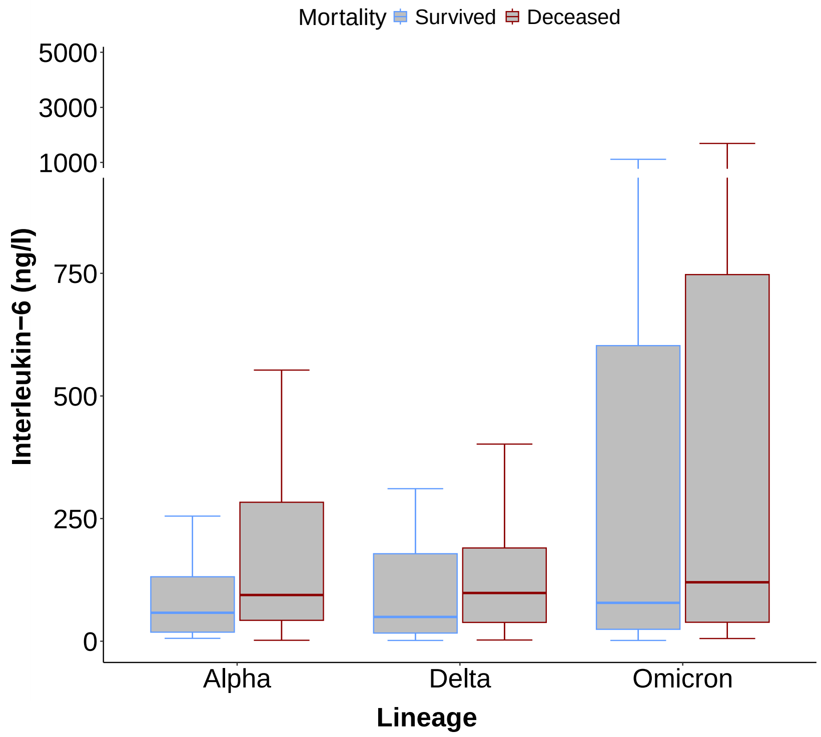

Figure S3. IL-6 levels in survived and deceased ICU patients of all lineages.

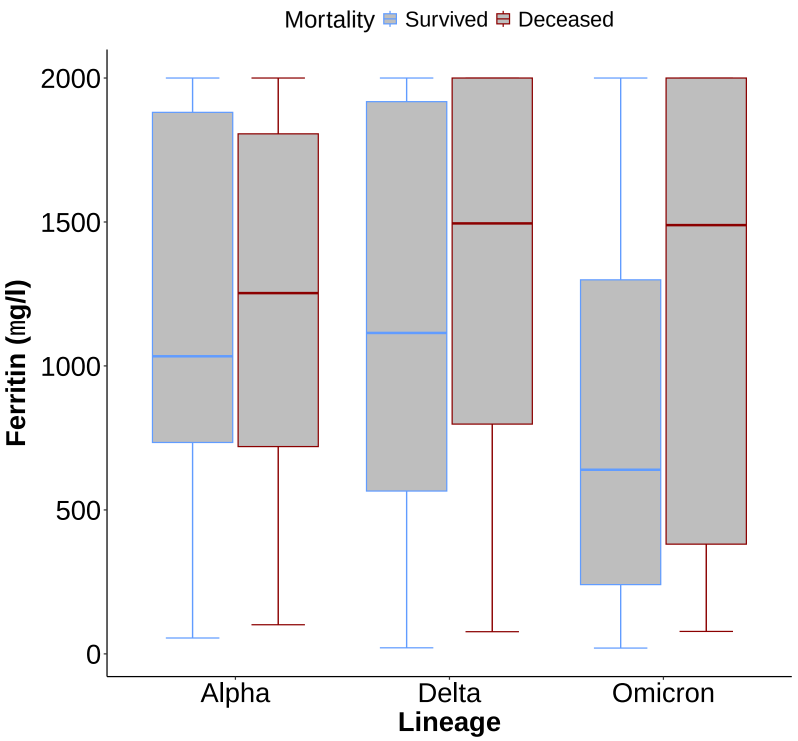

Figure S4. Ferritin levels in survived and deceased ICU patients of all lineages.

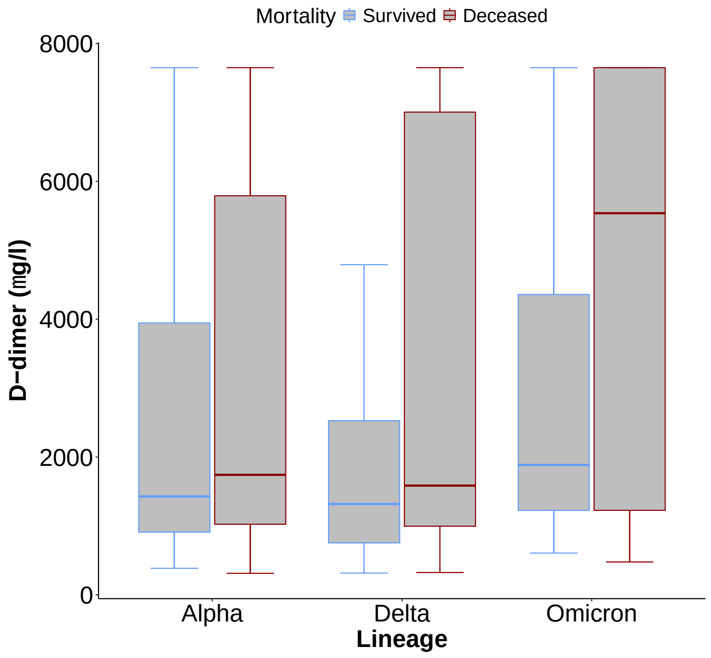

Figure S5. D-dimer levels in survived and deceased ICU patients of all lineages.

Table S7. Detailed characteristics of various clinical and laboratory parameters of ICU patients in Alpha, Delta, and Omicron lineages

	Alpha				Delta				Omicron			
	Overall	Survived	Deceased	p-value	Overall	Survived	Deceased	p-value	Overall	Survived	Deceased	p-value
**Interleukin-6**				**0.006**[1]				**0.008**[1]				0.391[1]
Mean (SD)	369 (922)	227 (708)	449 (1,018)		299 (814)	275 (810)	312 (818)		807 (1,546)	589 (1,261)	1,048 (1,803)	
Median (IQR)	69 (31, 214)	58 (18, 131)	94 (43, 283)		80 (27, 190)	49 (17, 178)	98 (38, 190)		85 (26, 678)	78 (24, 603)	120 (39, 747)	
Minimum; Maximum	2; 5,000	6; 5,000	2; 5,000		2; 5,000	2; 5,000	2; 5,000		2; 5,000	2; 5,000	5; 5,000	
N (% not missing)	149 (77%)	54 (81%)	95 (75%)		204 (89%)	70 (90%)	134 (88%)		59 (75%)	31 (76%)	28 (74%)	
NA	45	13	32		26	8	18		20	10	10	
**Ferritin**				0.649[1]				**0.009**[1]				**0.026**[1]
Mean (SD)	1,198 (602)	1,161 (629)	1,217 (591)		1,309 (638)	1,150 (666)	1,389 (611)		989 (741)	771 (637)	1,246 (782)	
Median (IQR)	1,158 (731, 1,862)	1,034 (734, 1,881)	1,253 (720, 1,806)		1,353 (720, 2,000)	1,115 (566, 1,918)	1,495 (798, 2,000)		819 (259, 1,889)	640 (241, 1,299)	1,489 (381, 2,000)	
Minimum; Maximum	55; 2,000	55; 2,000	101; 2,000		21; 2,000	21; 2,000	77; 2,000		20; 2,000	20; 2,000	78; 2,000	
N (% not missing)	124 (64%)	42 (63%)	82 (65%)		203 (88%)	68 (87%)	135 (89%)		63 (80%)	34 (83%)	29 (76%)	
NA	70	25	45		27	10	17		16	7	9	
**D-dimer**				0.157[1]				**0.016**[1]				0.057[1]
Mean (SD)	3,094 (2,718)	2,777 (2,634)	3,264 (2,758)		2,906 (2,694)	2,252 (2,255)	3,244 (2,844)		3,556 (2,748)	2,818 (2,214)	4,421 (3,076)	
Median (IQR)	1,570 (1,004, 5,661)	1,428 (910, 3,945)	1,742 (1,025, 5,792)		1,468 (959, 4,592)	1,318 (754, 2,527)	1,586 (996, 7,005)		2,308 (1,227, 6,687)	1,885 (1,227, 4,358)	5,538 (1,227, 7,650)	
Minimum; Maximum	312; 7,650	385; 7,650	312; 7,650		316; 7,650	316; 7,650	325; 7,650		477; 7,650	606; 7,650	477; 7,650	
N (% not missing)	175 (90%)	61 (91%)	114 (90%)		214 (93%)	73 (94%)	141 (93%)		76 (96%)	41 (100%)	35 (92%)	
NA	19	6	13		16	5	11		3	0	3	
**Lymphocyte count**				**0.001**[1]				0.169[1]				0.411[1]
Mean (SD)	0.70 (0.37)	0.81 (0.38)	0.65 (0.36)		0.86 (0.55)	0.91 (0.54)	0.84 (0.56)		0.72 (0.52)	0.71 (0.41)	0.73 (0.63)	

Median (IQR)	0.61 (0.40, 0.91)	0.76 (0.56, 1.08)	0.54 (0.38, 0.84)		0.74 (0.53, 1.06)	0.79 (0.58, 1.17)	0.70 (0.52, 1.02)		0.54 (0.38, 0.91)	0.67 (0.42, 0.94)	0.49 (0.38, 0.88)	
Minimum; Maximum	0.18; 2.29	0.18; 1.76	0.18; 2.29		0.13; 3.15	0.13; 3.15	0.14; 3.11		0.05; 3.10	0.05; 1.97	0.28; 3.10	
N (% not missing)	187 (96%)	63 (94%)	124 (98%)		212 (92%)	67 (86%)	145 (95%)		74 (94%)	38 (93%)	36 (95%)	
NA	7	4	3		18	11	7		5	3	2	

**Chest CTSS**				0.088[1]				0.056[1]				0.097[1]
Mean (SD)	18.8 (5.8)	18.3 (5.4)	19.0 (6.0)		18.8 (5.7)	17.6 (6.7)	19.5 (5.0)		12 (9)	10 (8)	13 (9)	
Median (IQR)	20.0 (17.0, 23.0)	19.0 (16.0, 22.0)	20.0 (17.0, 23.0)		20.0 (17.0, 23.0)	18.0 (16.0, 22.0)	20.0 (17.5, 23.0)		15 (0, 19)	12 (0, 17)	17 (3, 22)	
Minimum; Maximum	0.0; 25.0	0.0; 25.0	0.0; 25.0		0.0; 25.0	0.0; 25.0	0.0; 25.0		0; 25	0; 22	0; 25	
N (% not missing)	190 (98%)	65 (97%)	125 (98%)		208 (90%)	73 (94%)	135 (89%)		71 (90%)	36 (88%)	35 (92%)	
NA	4	2	2		22	5	17		8	5	3	

**P/F ratio**				0.055[1]				**<0.001**[1]				**0.009**[1]
Mean (SD)	73 (39)	81 (47)	68 (33)		68 (34)	80 (46)	62 (23)		99 (70)	113 (75)	82 (60)	
Median (IQR)	62 (52, 78)	65 (55, 90)	61 (51, 75)		60 (50, 71)	63 (55, 84)	57 (49, 67)		79 (54, 120)	85 (65, 132)	59 (48, 101)	
Minimum; Maximum	27; 322	31; 322	27; 307		27; 301	33; 301	27; 187		34; 390	34; 390	35; 357	
N (% not missing)	152 (78%)	51 (76%)	101 (80%)		194 (84%)	66 (85%)	128 (84%)		77 (97%)	41 (100%)	36 (95%)	
NA	42	16	26		36	12	24		2	0	2	

[1] Wilcoxon rank sum test

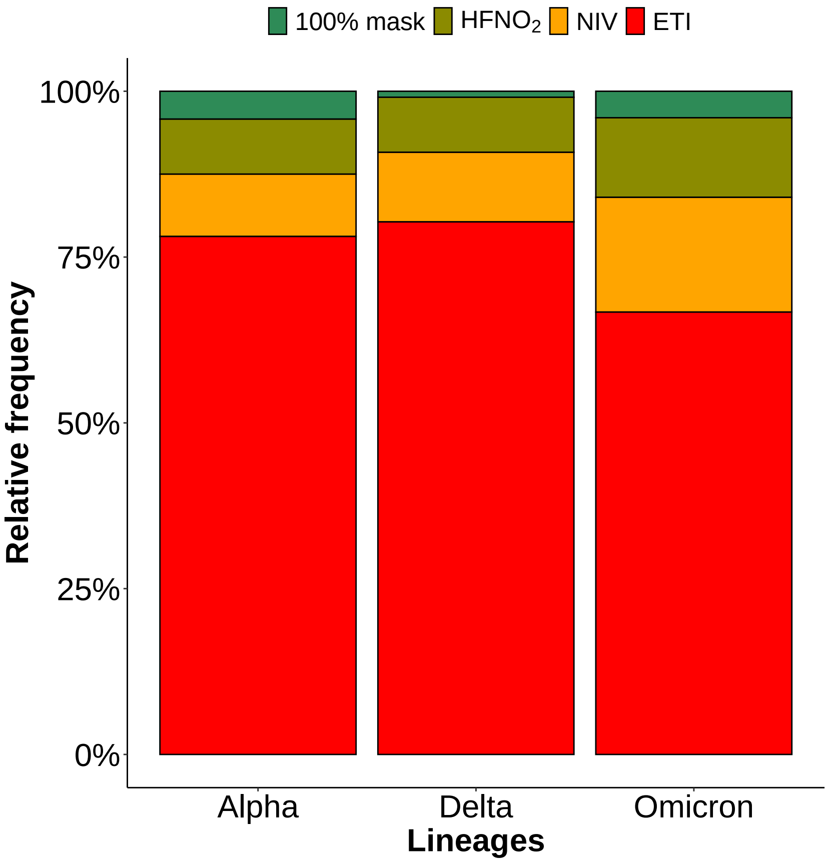

Figure S6. Relative frequency of oxygen supplementation types in ICU patients of all lineages

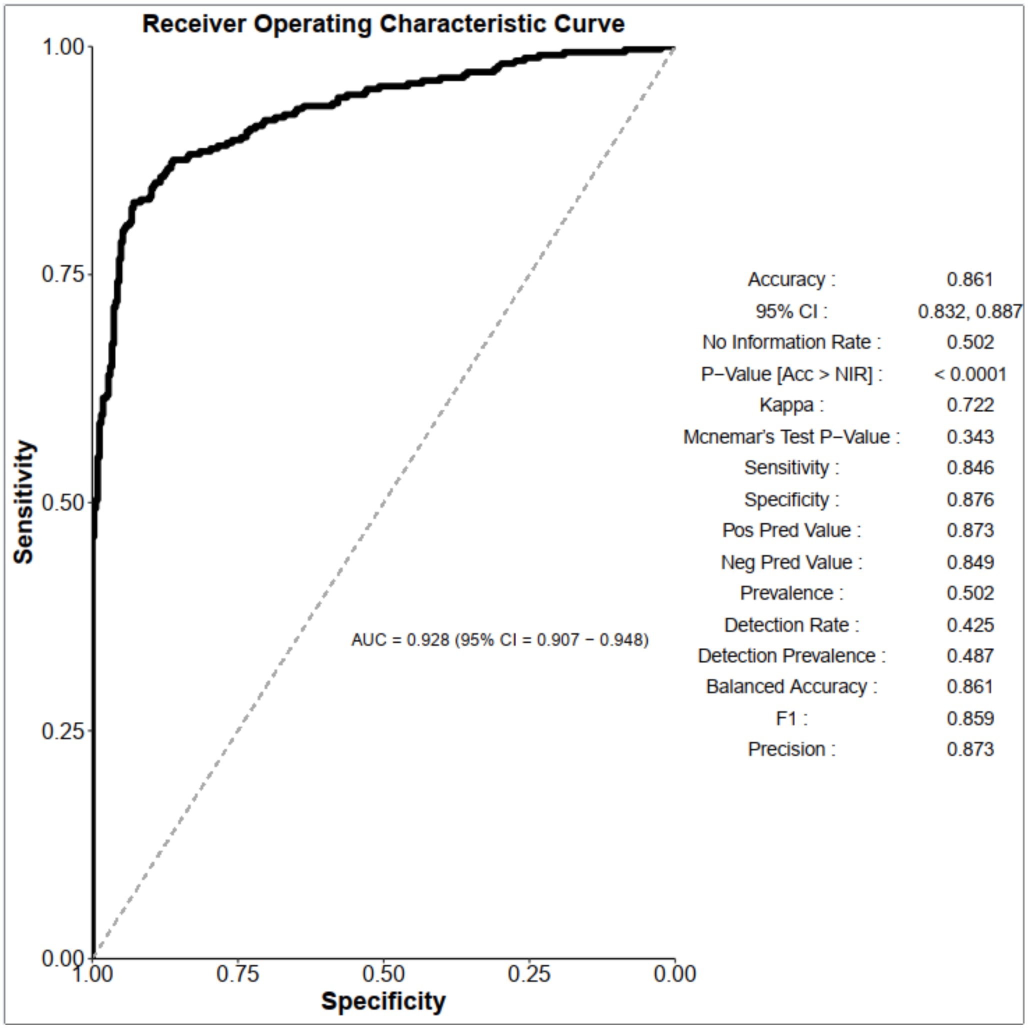

Figure S7. Training model performance metrics with the ROC curve

Table S8. Exact values of the top variables

	Deceased	Survived	MDA	MDG
Age	13.957409	58.34331	53.17803	36.36885
Chest CTSS	6.911023	61.65840	57.03918	32.43799
Days spent in ICU	8.487308	54.08310	48.77691	32.09919
P/F ratio	20.056242	82.96484	79.01285	53.47325
C Reactive Protein	3.711238	48.94962	44.00111	30.36394
D-dimer	4.292926	46.37849	41.37512	27.15413
PCT	1.696726	56.23356	47.60974	33.56495
Lymphocyte count	10.790269	62.52023	57.12910	39.57276
Leukocyte count	6.825290	59.10516	53.46046	37.97264

Table S9. Comparison of the original, imputed and balanced datasets

**Variables**	**Type of datasets**			**p-value**
	**Original**	**Imputed**	**Balanced**	
	N = 649 (32%)	N = 649 (32%)	N = 760 (37%)	
**Age**				0.482[1]
Mean (SD)	65 (13)	65 (13)	64 (13)	
Median (IQR)	66 (57, 74)	66 (57, 74)	65 (57, 74)	
**Chest CTSS**				0.096[1]
Mean (SD)	16 (7)	16 (7)	16 (7)	
Median (IQR)	18 (12, 22)	18 (12, 22)	18 (12, 21)	
NA	42	0	0	
**Days spent in ICU**				0.901[1]
Mean (SD)	12 (9)	12 (9)	12 (9)	
Median (IQR)	10 (6, 16)	10 (6, 16)	10 (6, 16)	
NA	1	0	0	
**P/F ratio**				0.093[1]
Mean (SD)	95 (81)	92 (75)	97 (79)	
Median (IQR)	65 (53, 100)	66 (56, 99)	67 (57, 106)	
NA	106	0	0	
**CRP**				0.591[1]
Mean (SD)	128 (87)	127 (86)	123 (85)	
Median (IQR)	114 (57, 185)	114 (58, 180)	111 (56, 171)	

NA	23	0	0	
**D-dimer**				0.587[1]
Mean (SD)	3,003 (2,705)	2,959 (2,525)	2,904 (2,502)	
Median (IQR)	1,517 (948, 5,404)	1,822 (1,011, 4,375)	1,842 (969, 4,358)	
NA	91	0	0	
**PCT**				0.252[1]
Mean (SD)	2.95 (11.66)	2.98 (11.22)	3.47 (13.27)	
Median (IQR)	0.33 (0.14, 0.86)	0.38 (0.15, 1.17)	0.34 (0.14, 1.08)	
NA	52	0	0	
**Lymphocyte count**				0.733[1]
Mean (SD)	0.83 (0.54)	0.83 (0.53)	0.82 (0.50)	
Median (IQR)	0.69 (0.45, 1.05)	0.71 (0.46, 1.03)	0.73 (0.49, 1.05)	
NA	32	0	0	
**Leukocyte count**				0.302[1]
Mean (SD)	10.2 (5.6)	10.2 (5.5)	9.8 (5.2)	
Median (IQR)	9.1 (6.3, 12.7)	9.2 (6.4, 12.7)	8.8 (6.2, 12.3)	
NA	8	0	0	
**Mortality, n (%)**				**<0.001**[2]
Deceased	382 (58.9%)	382 (58.9%)	382 (50.3%)	
Survived	267 (41.1%)	267 (41.1%)	378 (49.7%)	
1 Kruskal-Wallis rank sum test				
2 Pearson’s Chi-squared test				

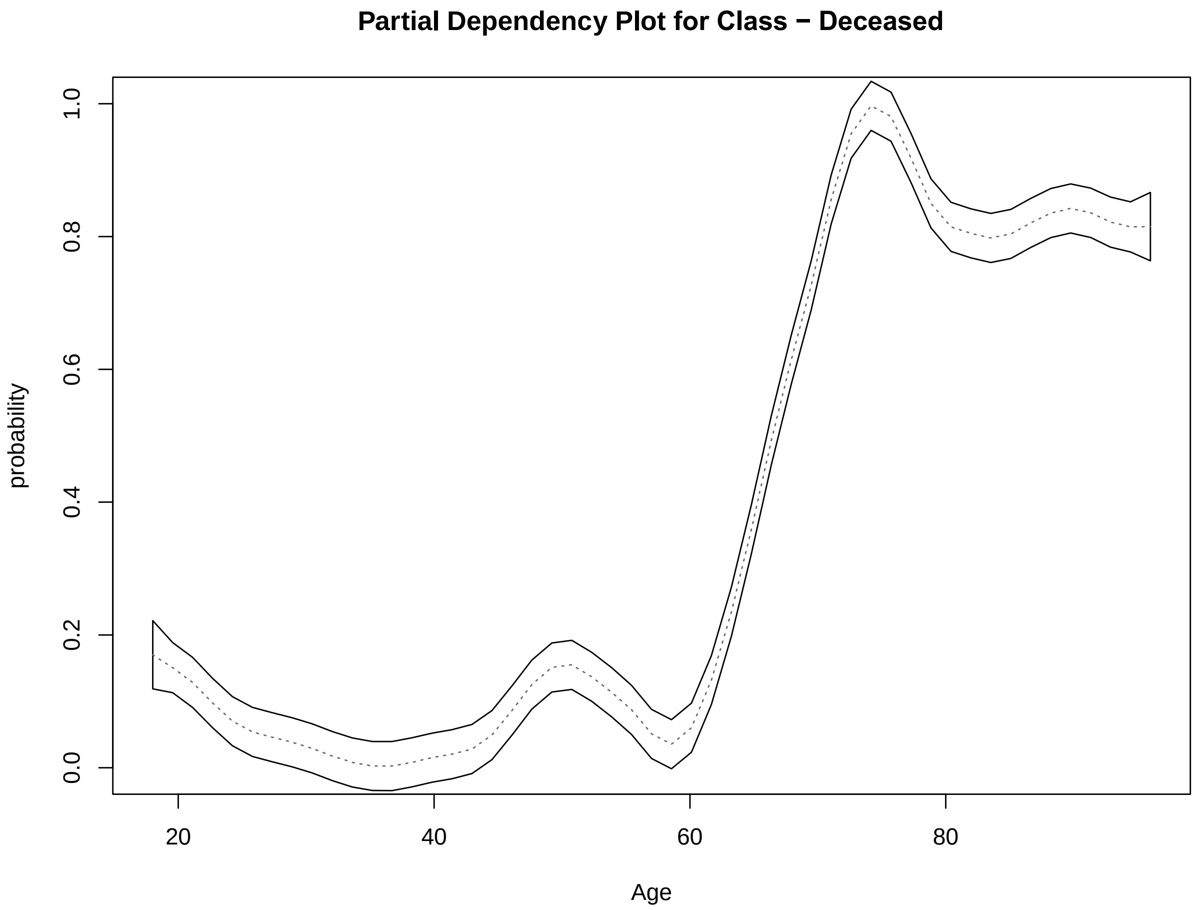

Figure S8. Partial dependency plot of age

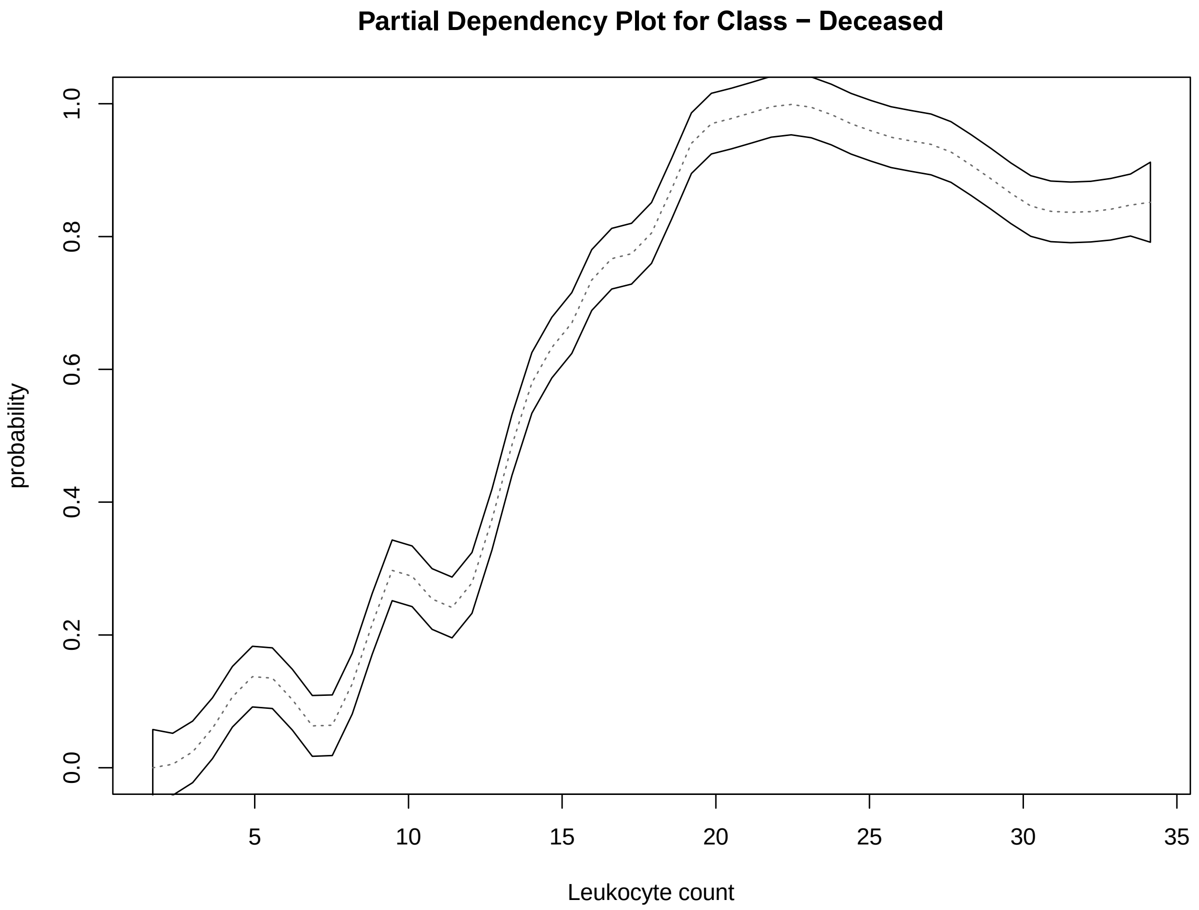

Figure S9. Partial dependency plot of leukocyte count

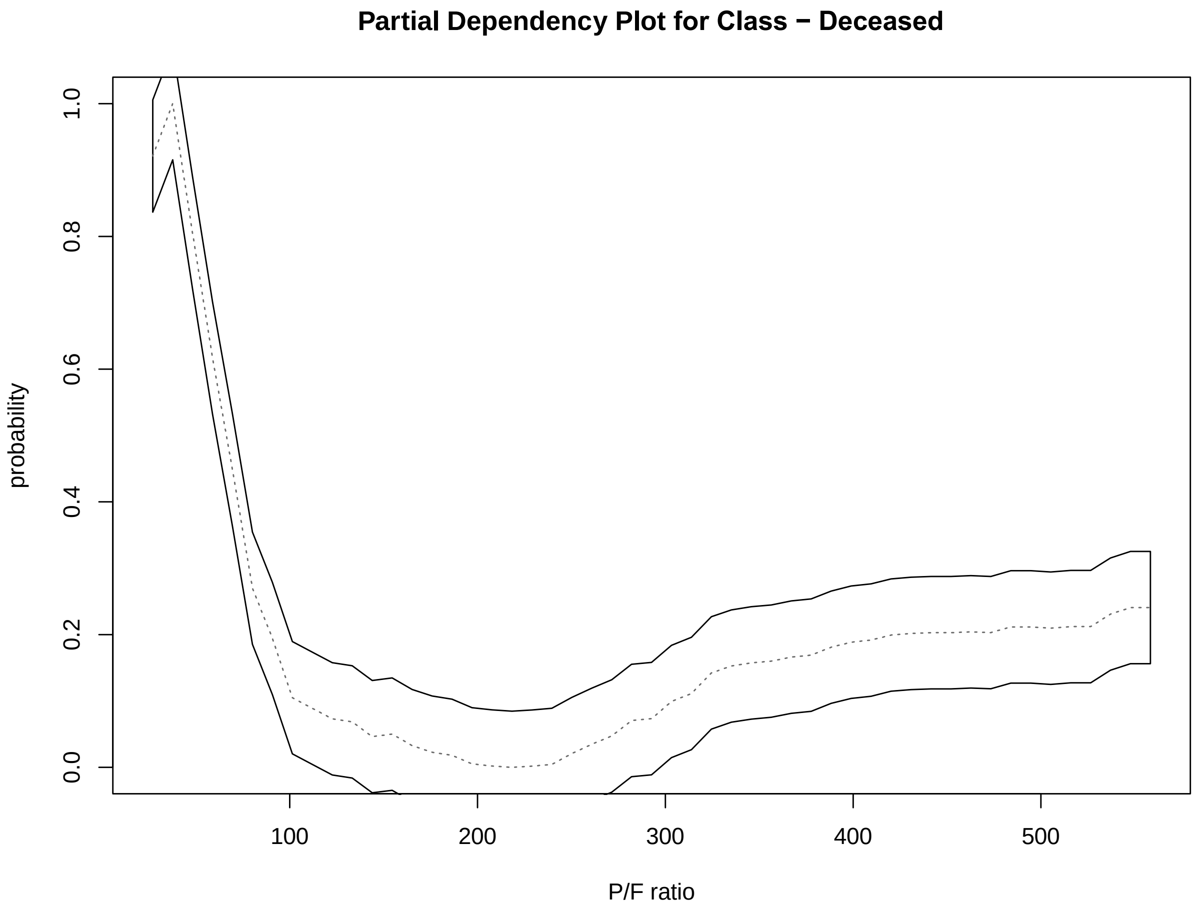

Figure S10. Partial dependency plot of P/F ratio

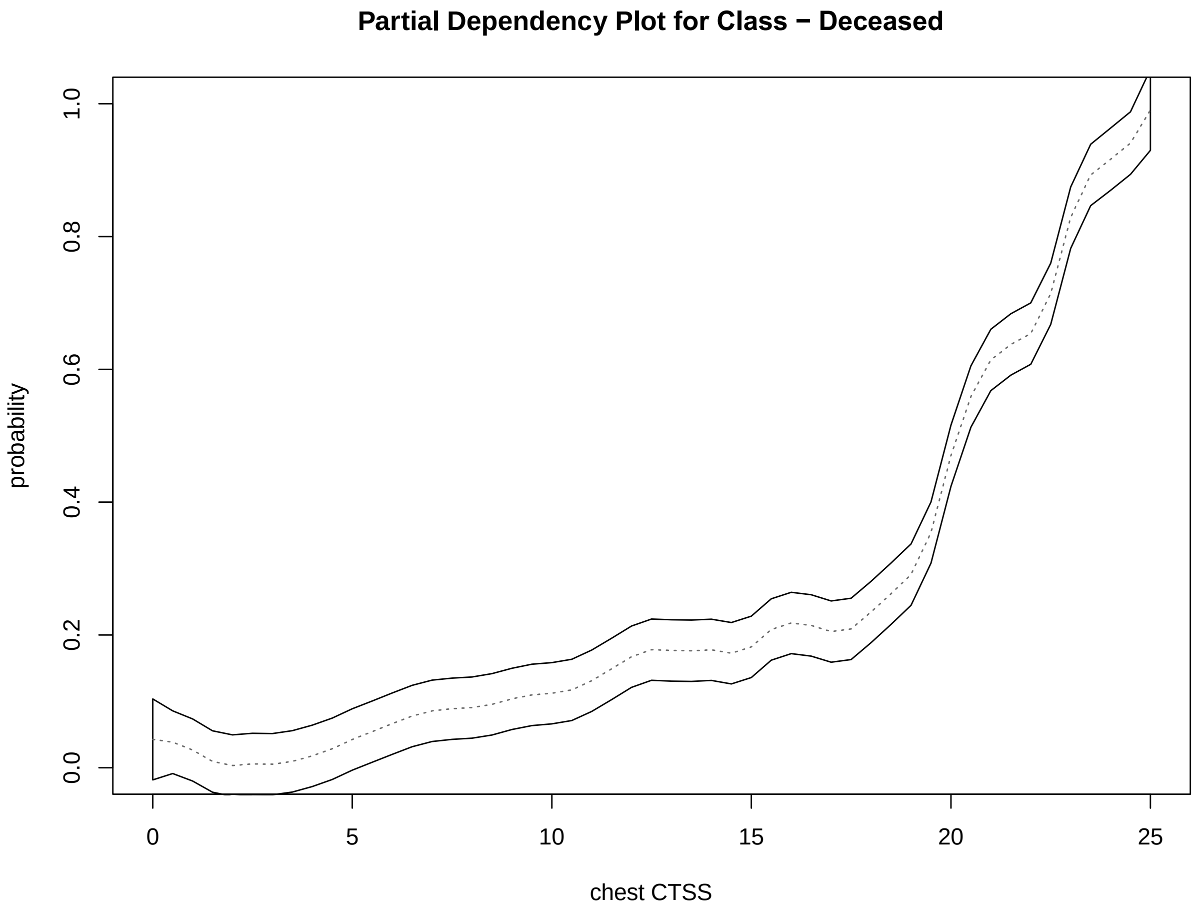

Figure S11. Partial dependency plot of chest CTSS

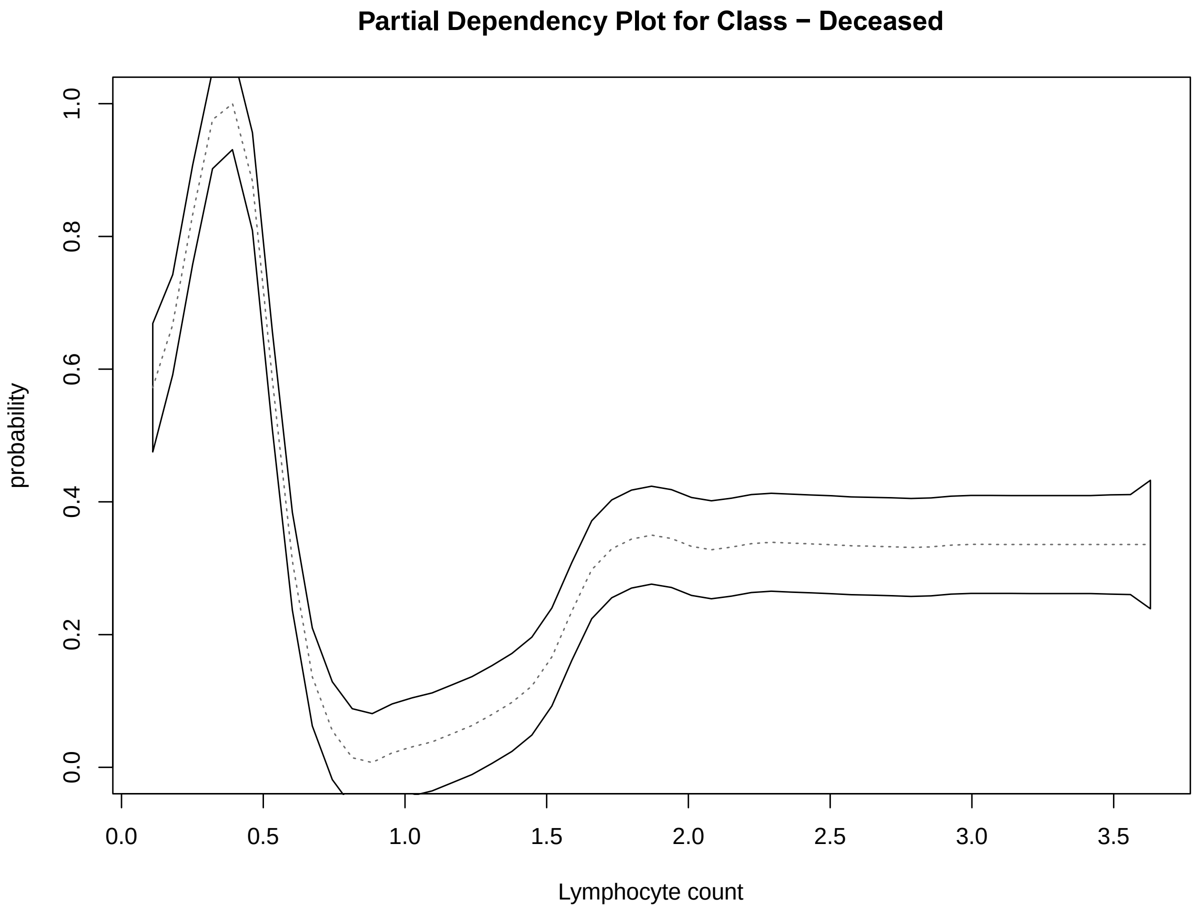

Figure S12. Partial dependency plot of lymphocyte count

Materials and methods – Supplementary

Nucleic acid extraction and reverse-transcription quantitative PCR

Nucleic acid is extracted from a 200µl specimen either manually or with the MagNaPure 96 automated nucleic acid extraction system (Roche, Mannheim, Germany). According to all the manufacturer's instructions and protocol, automated extraction was optimized using the MagNA Pure 96 DNA and Viral NA SV Kit (Cat No. 654358800, Roche). During manual nucleic acid extraction, the HighPure RNA isolation kit's (Cat No.11858882001, Roche) provided protocol was followed comprehensively. Both automated and manual nucleic acid extraction procedures included 5 µl of LightMix Modular EAV RNA extraction control (Cat No. 61090996, TIB Molbiol) to verify extraction and reverse transcription.

To detect the presence of SARS-CoV-2 RNA in the RT-qPCR analysis, three regions of the positive-sense single-stranded viral genome were targeted: conserved fragments of the sequences encoding the envelope protein (E-gene), (Cat No. 53077696), nucleocapsid protein (N-gene) (Cat No. 53077596), and RNA dependent RNA polymerase (RdRP-gene), (Cat No. 53077796).

PCR Master mixes were prepared to contain 0.5 µl of target-specific primer and probe mix, 4 µl of Real-Time ready Virus Master reaction buffer, 0.5 µl extraction control target-specific primer and probe mix, and 0.4 µl Real Time ready Virus Master RT enzyme (Cat No. 05992877001, Roche), 10.4 µl PCR grade water and 5 µl of the RNA sample. For negative controls, we prepared a Master mix where template RNA was substituted with PCR grade water. PCR amplification was carried out in Cobas Z 480 PCR systems with the following thermic conditions [reverse transcription one cycle: 55°C for 5 min; enzyme activation one cycle: 95 °C for 5 min; amplification 45 cycles {95 °C for 5s, 60 °C for 15s, 72 °C for 15s].

Cycle threshold values were calculated with Exor 4.0. Cobas Z 480 (Roche Diagnostics). Results were analyzed, and fluorescence data were evaluated in Exor 4.0 software.

Sampling procedure

Representative clinical isolates of 503 patients were used to characterize SARS-CoV-2 lineages in the timeframe of the study basic up on having positive results of RT-qPCR. For every single sample, two nasopharyngeal swabs are collected from patients suspected of having a COVID-19 infection by medical doctors and/or trained nurses in the different wards of ICUs of the Clinical Center of the Pécs University. First, the nasopharyngeal swabs were washed into the sample collection tube containing the Virus Transport Medium (VTM) (CE certified, Biolabs Ltd, Hungary), and swabs were broken at the designed grooves, and later the remaining holding part of the swabs was removed and safely discarded. Then, sample collection tubes are individually wrapped in a sterile double-wall plastic bag and transferred to the laboratory at 4 °C for RNA extraction and isolation.

Whole Genome Sequencing

WGS is performed by the Bioinformatics Core Facility at Szentágothai Research Centre of the University of Pécs. Viral RNA was extracted with Omega MagBind Viral DNA/RNA kits (Omega Bio-Tek, Cat. No. M6246-03) on Hamilton Starlet Automated Liquid Handling System (Hamilton, Germany) according to the manufacturer's instructions. Isolated RNA was qualified by Qubit 3.0 (Invitrogen). Library preparation was conducted using Twist EF Library Prep 2.0 (Twist Bioscience), and the viral genome capture was performed by Twist Comprehensive Panel (Twist Bioscience). The captured libraries were checked for quality using TapeStation 4200 Agilent and Qubit 3.0. The libraries were sequenced on NovaSeq 6000 (Illumina) for 2x150 paired-end reads. Raw sequences were quality-checked with FastQC [1]. During the next step, the readouts were trimmed with Trimmomatic [2]. The remaining high-quality readings were mapped against the MN908947 reference sequence. For the mapping, Geneious (Geneious Prime 2020.0.3) default algorithm was applied with the default parameters. Samtools mpileup [3], and varscan mpileuo2cns [4] were

used to determine the variants, and these variants were used to create the consensus sequence; the minimum coverage was set to 15.

References:

1. Andrews, S. FastQC: A Quality Control Tool for High Throughput Sequence Data. https://www.bioinformatics.babraham.ac.uk/projects/fastqc/ (2010).
2. Bolger, A. M., Lohse, M. & Usadel, B. Trimmomatic: a flexible trimmer for Illumina sequence data. *Bioinformatics* **30**, 2114–20 (2014).
3. Danecek, P. *et al.* Twelve years of SAMtools and BCFtools. *Gigascience* **10**, (2021).
4. Koboldt, D. C. *et al.* VarScan: variant detection in massively parallel sequencing of individual and pooled samples. *Bioinformatics* **25**, 2283–5 (2009).
